# Hydraulically‐vulnerable trees survive on deep‐water access during droughts in a tropical forest

**DOI:** 10.1111/nph.17464

**Published:** 2021-07-02

**Authors:** Rutuja Chitra‐Tarak, Chonggang Xu, Salomón Aguilar, Kristina J. Anderson‐Teixeira, Jeff Chambers, Matteo Detto, Boris Faybishenko, Rosie A. Fisher, Ryan G. Knox, Charles D. Koven, Lara M Kueppers, Nobert Kunert, Stefan J. Kupers, Nate G. McDowell, Brent D. Newman, Steven R. Paton, Rolando Pérez, Laurent Ruiz, Lawren Sack, Jeffrey M. Warren, Brett T. Wolfe, Cynthia Wright, S. Joseph Wright, Joseph Zailaa, Sean M. McMahon

**Affiliations:** ^1^ Los Alamos National Laboratory Earth and Environmental Sciences Division (EES‐14) MS J495 Los Alamos NM 87545‐1663 USA; ^2^ Smithsonian Environmental Research Center 647 Contees Wharf Road Edgewater MD 21037‐0028 USA; ^3^ Smithsonian Tropical Research Institute Balboa Apartado 0843–03092 Republic of Panama; ^4^ Conservation Ecology Center Smithsonian Conservation Biology Institute Front Royal VA 22630 USA; ^5^ Lawrence Berkeley National Laboratory Climate and Ecosystem Sciences Division Berkeley CA 94720 USA; ^6^ Ecology and Evolutionary Biology Princeton University Princeton NJ 08544 USA; ^7^ Climate and Global Dynamics Division National Center for Atmospheric Research Boulder CO 80305 USA; ^8^ Laboratoire Évolution & Diversité Biologique CNRS:UMR 5174 Université Paul Sabatier Toulouse 31062 France; ^9^ Energy and Resources Group University of California Berkeley 310 Barrows Hall #3050 Berkeley CA 94720 USA; ^10^ Department of Integrative Biology and Biodiversity Research Institute of Botany University of Natural Resources and Life Sciences Vienna Gregor‐Mendel‐Str 33 Wien A‐1190 Austria; ^11^ Computational Forest Ecology German Centre for Integrative Biodiversity Research (iDiv) Halle‐Jena‐Leipzig Leipzig Saxony 04103 Germany; ^12^ Atmospheric Sciences and Global Change Division Pacific Northwest National Lab PO Box 999 Richland WA 99352 USA; ^13^ School of Biological Sciences Washington State University PO Box 644236, Pullman WA 99164‐4236 USA; ^14^ Indo‐French Cell for Water Sciences Indian Institute of Science Bangalore 560012 India; ^15^ UMR GET IRD CNRS UPS Toulouse 31700 France; ^16^ Institut Agro UMR SAS INRAE Rennes 35042 France; ^17^ Ecology and Evolutionary Biology University of California Los Angeles 612 Charles E. Young Drive South Los Angeles CA 90095 USA; ^18^ Oak Ridge National Laboratory Environmental Sciences Division Oak Ridge TN 37831 USA; ^19^ School of Renewable Natural Resources Louisiana State University Agricultural Center Baton Rouge LA 70803 USA; ^20^ Biological Sciences Department California State University Los Angeles Los Angeles CA 90032 USA

**Keywords:** deep‐water access, drought tolerance, drought‐induced mortality, hydraulic vulnerability and safety margins, hydrological droughts, rooting depths, safety‐efficiency trade‐off, tropical forest

## Abstract

- Deep‐water access is arguably the most effective, but under‐studied, mechanism that plants employ to survive during drought. Vulnerability to embolism and hydraulic safety margins can predict mortality risk at given levels of dehydration, but deep‐water access may delay plant dehydration. Here, we tested the role of deep‐water access in enabling survival within a diverse tropical forest community in Panama using a novel data‐model approach.
- We inversely estimated the effective rooting depth (ERD, as the average depth of water extraction), for 29 canopy species by linking diameter growth dynamics (1990–2015) to vapor pressure deficit, water potentials in the whole‐soil column, and leaf hydraulic vulnerability curves. We validated ERD estimates against existing isotopic data of potential water‐access depths.
- Across species, deeper ERD was associated with higher maximum stem hydraulic conductivity, greater vulnerability to xylem embolism, narrower safety margins, and lower mortality rates during extreme droughts over 35 years (1981–2015) among evergreen species. Species exposure to water stress declined with deeper ERD indicating that trees compensate for water stress‐related mortality risk through deep‐water access.
- The role of deep‐water access in mitigating mortality of hydraulically‐vulnerable trees has important implications for our predictive understanding of forest dynamics under current and future climates.

## Introduction

Drought‐induced mortality in tropical forests may have significant global implications. Tropical forests play a disproportionately large role in the global carbon and energy cycles (Bonan, 2008), and support half of global biodiversity (Wright, 2005), but face a threat from intensifying droughts (Malhi *et* *al*., 2009; Doughty *et* *al*., 2015; Xu *et* *al*., 2019). Tropical forests are considered an especially drought‐vulnerable biome given the combination of climate risk and vegetation sensitivity (Meir *et* *al*., 2015). Still, mortality events in the tropics are rarely as large as those in temperate and boreal zones (McDowell *et* *al*., 2018a), leading to questions regarding the role of large trait‐diversity and hydraulic strategies in mitigating mortality events. Furthermore, mortality rates are increasing in some tropical regions (Brienen *et* *al*., 2015; Hubau *et* *al*., 2020) and widespread drought‐induced tree mortality has occurred across the tropics for specific functional groups (Phillips *et* *al*., 2010; Hilker *et* *al*., 2014; Bennett *et* *al*., 2015; Chitra‐Tarak *et* *al*., 2018). State‐of‐the‐art dynamic global vegetation models (DGVMs) struggle to capture these drought‐induced vegetation dynamics in the tropics (Galbraith *et* *al*., 2010; Powell *et* *al*., 2013, 2018), because underlying mechanisms of drought tolerance are not fully understood nor quantified.

Plants rely on a variety of structural and functional mechanisms to avoid or tolerate a drought, from deep‐water access, increased root production, hydraulic redistribution, embolism resistance, adjustment of leaf area (deciduousness) to change in leaf angle, reductions in stomatal conductance, upregulation of aquaporins, osmotic regulation and stem water storage capacitance (McDowell *et* *al*., 2008). Deep‐water access is arguably the most effective, yet under‐studied mechanism. As plant‐available water varies with depth, trees within the same forest with different rooting depths, depending on species and size (Meinzer *et* *al*., 1999; Chitra‐Tarak *et* *al*., 2018; Brum *et* *al*., 2019), differ in their experience during a drought, and thus in their growth and mortality responses (Chitra‐Tarak *et* *al*., 2018). A key bottleneck in community‐wide testing of this mechanism has been a lack of data in both trees’ rooting or water‐sourcing depths, and plant‐available soil water at those depths. A recent meta‐analysis documented maximum rooting depths for 318 tree species, that is, < 0.5% of > 60 000 tree species in the World (Beech *et* *al*., 2017; Fan *et* *al*., 2017). Furthermore, only a small fraction of those are tropical, even though > 90% of the World’s tree diversity resides in the tropics (Slik *et* *al*., 2015). The use of stable isotopes of water as a tracer provides an indirect measure of water sourcing depths by matching the isotopic value in xylem water to those in soil pore water at different depths. However, such data are rare (Evaristo *et* *al*., 2016). DNA barcoding of roots (Jones *et* *al*., 2011) may be used to estimate species‐specific rooting depths or profiles, but DNA barcode libraries for tropical forests are still under development, and the method may be cost‐prohibitive for the extent of sampling required. In general, community‐scale data collection for rooting or water‐sourcing depths is a formidable challenge in species‐rich tropical forests.

Characterizing the essential constraints to model species‐rich communities (Wright *et* *al*., 2010; Christoffersen *et* *al*., 2016; Maréchaux & Chave, 2017; Bartlett *et* *al*., 2019; Koven *et* *al*., 2020; Lu *et* *al*., 2020) entails identifying the topography of plant trait trade‐offs in different environments, and how these relate to demographic rates (growth, recruitment and mortality). Significant efforts have been invested into identifying and linking universal drought indices, aboveground traits and demographic rates. Such efforts have found correlations between vulnerability to embolism and hydraulic safety margins and mortality (Anderegg *et* *al*., 2016), although numerous counter examples also exist (Hoffmann *et* *al*., 2011; Paddock III *et al*., 2013; Nardini *et* *al*., 2015; Venturas *et* *al*., 2016; Johnson *et* *al*., 2018). Deep roots may mitigate hydraulic vulnerability (Brum *et* *al*., 2019) and mortality risk, in particular during hydrological (rather than meteorological) droughts. Nonetheless, the interaction between rooting depths, aboveground hydraulic traits, hydrological droughts quantified over the whole soil‐column and mortality outcomes are hardly studied.

In this paper, we estimate plant‐available water in the whole‐soil column in a tropical forest, and inversely estimate rooting depths of co‐occurring tree species from their growth responses, with a series of model calibrations and validation. We evaluate how rooting depth is linked to aboveground hydraulic traits and mortality rates through seven census intervals over a 35‐year period that experienced El Niño droughts of a variety of intensity, frequency and duration (Condit, 2017; Detto *et* *al*., 2018). Our hypotheses are that (1) deep‐rooted trees have hydraulic traits associated with rapid water transport but cavitation‐vulnerable xylem resulting from greater and more reliable water availability at depth (see Tables 1 and 2); and that, (2) deep‐rooted species have lower mortality rates during droughts resulting from a lower exposure to water stress compared to shallow‐rooted species. To our knowledge this is the first study to test for a mechanistic link between plant‐available water in the whole‐soil column, tree above‐ and belowground hydraulic traits, and multidecadal mortality outcomes for a species‐rich tropical forest.

**Table 1:** The symbol, definition and units of key traits used in the simulations and analyses.

Symbol	Definition	Units
$\Psi_{\text{soil},z}$	Soil water potential at depth z	MPa
Ψ_leaf_, Ψ_stem_	Water potential of leaf, or stem, respectively	MPa
Ψ_tlp_	Bulk leaf turgor loss point, the Ψ_leaf_ where turgor potential = 0	MPa
Ψ_crit_ or Ψ_20,leaf_	Ψ_leaf_ at 20% loss of leaf conductance	MPa
Ψ_88,stem_	Ψ_stem_ at 88% loss of stem conductivity	MPa
Ψ_min_	Seasonal minimum leaf water potential, the most negative Ψ_leaf_ measured at midday in the dry season	MPa
Ψ_min_ ‐ Ψ_88,stem_	Aboveground hydraulic safety margin	MPa
*K*_leaf_	Leaf‐area specific hydraulic conductance of leaf	mmol m^−2^ s^−1^ MPa^−1^
*K*_max,leaf_	Maximum leaf area‐specific hydraulic conductance of leaf	mmol m^−2^ s^−1^ MPa^−1^
*K*_max,stem_	Maximum stem area‐specific hydraulic conductivity of stem	kg m^−1^ s^−1^ MPa^−1^
*FLC*_leaf_	Ratio between current and maximum leaf‐area specific hydraulic conductance of leaf	‐
WSG	Wood specific gravity	g cm^−3^
LMA	Leaf mass per unit area	g m^−2^
δ^2^H_xylem_	δ^2^H of tree xylem sap	‰

**Table 2:** Hypotheses for association between effective rooting depth (ERD) and aboveground hydraulic traits.

Variable	Deeper ERD	Shallower ERD
*K*_max,stem_	Higher	Lower
Ψ_88,stem_	Less negative	More negative
Ψ_tlp_	Less negative	More negative
Ψ_min_–Ψ_88,stem_	Narrower	Wider

## Materials and Methods

**Fig. 1:**
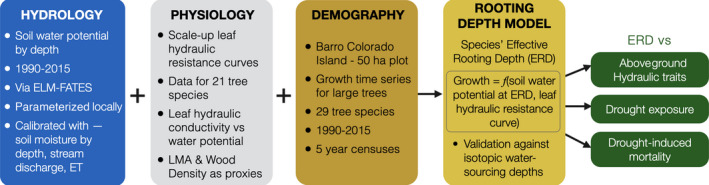
A schematic diagram outlining the methods workflow, which combines hydrology, physiology and demography of the tropical forest at Barro Colorado Island to inform the inverse model for rooting depths; validates it, and tests hypotheses pertaining to relationship of rooting depths with aboveground hydraulic traits, drought exposure, and drought‐induced mortality. ELM‐FATES, Energy Exascale Earth System Land Model coupled with the Functionally Assembled Terrestrial Ecosystem Simulator; ET, evapotranspiration; LMA, leaf mass per unit area.

This work combines hydrology, physiology and demography of a tropical forest at Barro Colorado Island, Panama, to inform the inverse model for rooting depths; validates the model, and tests hypotheses pertaining to relationship of rooting depths with aboveground hydraulic traits, drought exposure and mortality (Fig. 1). We defined species‐specific effective rooting depths (ERD) as the depth at which the growth factor determined by soil water potential and leaf hydraulic traits best explained species’ growth dynamics over 25 years. Developing a novel, empirical inverse model, we estimated ERDs for large trees of 29 species. The ERD model incorporated the impact of atmospheric and hydrological drought on growth, and was constrained with species‐specific leaf vulnerability curves. For the latter, we used existing data for eight species and developed trait‐based proxies for the rest (based on data for a total of 21 species). We obtained the daily dynamics of soil water potential in the whole‐soil column (≤ 13 m) over the 25 years by locally parameterizing a 1D hydrological water balance of the forest within a land surface model for an average vegetation type. The water balance was calibrated on available measurements, in particular continuous soil moisture data in three surface layers (over the first meter of soil), stream discharge and evapotranspiration.

We validated our ERD estimates against existing stable hydrogen isotope ratios (δ ^2^H_xylem_) for a subset of six tree species as independent observations. We evaluated whether ERD was associated with aboveground hydraulic traits, sourcing the latter from a set of rare datasets for six to seven species that overlapped with our assessment of ERD. We tested the relationship of ERD with xylem vulnerability to embolism, branch hydraulic safety margins, leaf turgor loss point and maximum stem‐specific hydraulic conductivity. We analyzed whether ERD is correlated with mortality dynamics of large trees for evergreen and deciduous species over 35 years marked by several El Niño events. Finally, to test whether ERD explained risk of drought‐induced mortality, we analysed species‐level exposure to water stress.

### Study site description

We conducted this study at Barro Colorado Island (BCI), Panama. The entire island is forested and classified as a tropical moist forest in the Holdridge Life Zone system. Long‐term hydrological monitoring at BCI began in 1972, whereas demographical monitoring began with the 50 ha ForestGEO plot establishment in 1981–82 (Condit, 1998; Hubbell *et* *al*., 1999; Anderson‐Teixeira *et* *al*., 2014). Rainfall at BCI is seasonal with a mean annual total of 2627 mm (± 516 SD; 1985–2019) and a pronounced dry season from mid‐December through April with < 100 mm of rainfall per month (Paton, 2019a, 2020). In the 50 ha (1000 m × 500 m) old growth forest plot, all stems ≥ 1 cm diameter at breast height (dbh) were mapped, tagged with a unique number, identified to species and measured every five years through 1985–2015 for growth, mortality as well as recruitment of new stems into the 1‐cm dbh size class (Condit, 2017). This inventory represents 321 woody species, 28% of which are at least partly dry‐season deciduous (Condit *et* *al*., 2000). The plot elevation is 120–160 m above sea level, and thus the elevation range is only 40 m (Harms *et* *al*., 2001). Soils are homogeneous with red light clays accounting for 72% of the plot (Baillie *et* *al*., 2007). The topsoil field texture is silty clay loam that gradually fines to silty clay in the subsoil. Soil is mostly free draining, but restricted subsoil permeability gives rise to temporary wet season ponding. Detailed descriptions of the climate, geology, flora and fauna of BCI can be found elsewhere (Croat, 1978; Leigh *et* *al*., 1982; Gentry, 1990).

### Model for ERD

#### ERD model description

Roots could impact tree growth through two factors: (1) water uptake; and (2) nutrient uptake. Because our study was situated in an old‐growth tropical forest, we assumed that nutrients are mainly concentrated in the shallow soil layers derived from litter decomposition, and that rooting depths do not substantially affect nutrient uptake. Because our study site has a dry season, we expected that rooting depth is a key factor affecting tree growth. We defined species‐specific effective rooting depth (ERD) as the depth at which a soil moisture growth limitation factor, B, determined by the soil water potential and hydraulic traits best explained species’ diameter growth dynamics. B is used to approximate the amount of stomatal closure due to water stress in the soil. In this study, we used the fractional loss of hydraulic conductivity of the leaf (FLC_leaf_) to estimate B. FLC_leaf_ is calculated as a fraction of maximum leaf conductivity $K_{\text{max,leaf}}$,

(Eqn 1) $${\text{FLC}}_{\text{leaf}}=\frac{K_{\text{leaf}}}{K_{\text{max,leaf}}}$$

where K_leaf_, current leaf hydraulic conductance; $K_{\text{max,leaf}}$, maximum leaf hydraulic conductance (see Table 1 for trait symbols and their definitions). The dynamics of K_leaf_ are estimated from the species‐specific relationship between K_leaf_ vs Ψ_leaf_, referred to as the leaf hydraulic vulnerability curve (Sack & Scoffoni, 2012). The vulnerability to loss of hydraulic conductivity arises not only from embolism, but also from extra‐xylem processes (Scoffoni & Sack, 2017). Because pre‐dawn Ψ_leaf_ generally approaches Ψ_soil_, this allowed us to substitute Ψ_leaf_ with Ψ_soil_ in leaf hydraulic vulnerability curves for each species s – defined below in Eqn 4 using species‐specific parameters $A_{s}$ and $B_{s}$ – to predict maximum diurnal $K_{\text{leaf},s,i}$ for each day i,

(Eqn 2) $$K_{\text{leaf},\mathrm{s,i}}=A_{s}e^{‐B_{s}\Psi_{\text{soil,}i}}$$

$K_{\text{max,leaf},s}$ was obtained as the maximum value of $K_{\text{leaf,s}}$ using Eqn 2. K_leaf_ is strongly related to photosynthetic capacity (Brodribb *et* *al*., 2002). Because we were interested in relating K_leaf_ to 5‐year average diameter growth observations, we ignored diurnal dynamics of K_leaf_.

Many other intrinsic and extrinsic factors limit plant growth, including soil moisture, vapor pressure deficit (VPD), radiation, leaf area seasonality (Brodribb *et* *al*., 2002; Lawrence *et* *al*., 2019). VPD affects growth nonlinearly, with growth increasing with VPD up to a VPD threshold, then decreasing as leaf pores (stomata) close, reducing water uptake (Yang *et* *al*., 2019; Grossiord *et* *al*., 2020). To account for the nonlinear impact of VPD on growth, we used predicted gross primary productivity (GPP; hereafter, $\hat{\text{VPD}}$) from a locally derived polynomial relationship between GPP and VPD (Supporting Information Dataset S1; Fig. S1). Apart from stomatal control, leaf deciduousness may further limit water uptake and growth.

We therefore tested alternate structures of empirical growth models (Methods S1, Eqns S1–S6, including Eqn S3 or Eqn 3), or inverse models of ERD, in which we regressed species‐specific growth against multiplicative or additive effects of one or more growth factors calculated daily and averaged over 5‐yearly censuses: $\hat{\text{VPD}}$, ${\text{FLC}}_{\text{leaf}}$ and the leaf area index (LAI). Incorporation of radiation did not improve model‐fitting, possibly due to the coincidence of higher temperature, higher radiation and lower humidity during the dry season at BCI.

The best empirical growth model, or inverse ERD model, structure that we found (see model validation and selection below) to describe daily average tree growth $\hat{G}$ for species s in the census interval t is described as follows, in which $K_{\text{leaf}}$, and thus $FLC_{\text{leaf}}$, is driven by soil water dynamics at z:

(Eqn 3) $${\hat{G}}_{s,t|z}=\beta_{0,s|z}+\beta_{1,s|z}\left(\frac{1}{n_{t}}\sum_{i=1}^{n_{t}}{\text{FLC}}_{\text{leaf},s,i|z}^{*}{\hat{\text{VPD}}}_{i}^{*}\right)+\epsilon_{s,t|z}$$

where |, conditionals; *n_t_*, total number of days in census interval t; * indicates that the variable has been standardized to range between 0 and 1 (within species for FLC_leaf_); $\beta_{0}$ and $\beta_{1}$, model coefficients; ϵ, model error term. See Methods S1 for all of the alternate model structures tested (Eqns S1–S6).

We evaluated different model structures and for each we estimated species ERD as the depth z at which soil water dynamics ($\Psi_{\text{soil,}z}$) best explained observed dynamics of growth G (see below) via modeled growth $\hat{G}$. Our growth model, or inverse ERD model, does not explicitly use rooting profiles, but identifies soil water dynamics at a single depth z as the central tendency that influences the observed growth dynamics the most. We modeled multiple hydrological realizations of soil water‐potential dynamics (see below). Incorporating this uncertainty, we defined species ERD as the median (± SE) of best‐fit depths across all hydrological realizations for soil water dynamics (${\text{Ψ}}_{\text{soil},z}$) (Eqn S7). See Methods S2 for statistics for identifying best‐fit ERD.

#### Growth data

For diameter growth estimates, to minimize the effect of light variation among trees, we selected only large trees (≥ 30 cm diameter at breast height (dbh)) in the 50‐ha plot and also species whose maximum height was ≥ 30 m (hereafter, canopy species) and thus are likely to be fully exposed to the sun. Calculating individual tree growth rates across six 5‐yearly censuses (1990–2015; Condit *et* *al*., 2019; Condit, 2019), removing outliers, obtaining residuals from a dbh model of growth to account for the size effect on growth (Methods S3), we estimated species s growth time series $G_{s}$ (cm yr^−1^) as the median of standardized dbh model residuals, for only those species (n = 29) with complete records for at least three trees (median 10, maximum 111 trees per species).

#### Leaf hydraulic vulnerability curves

We obtained leaf hydraulic vulnerability curves ($K_{\text{leaf}}$ vs Ψ_leaf_) for adult trees of 21 common species at BCI from J. Zailaa *et* *al*. (unpublished; see Dataset S2 for brief description of methods) described as:

(Eqn 4) $$K_{\text{leaf},s}=A_{s}e^{‐B_{s}\Psi_{{\text{leaf}}_{s}}}+\epsilon_{s}$$

where A and B, fitted species‐specific parameters; $\epsilon_{s}$, error term. These 21 species included eight of the 29 species selected for ERD estimation. For the remaining 22 species, we obtained vulnerability curves using trait‐based proxies. We identified scaling relationships between fitted parameters A and B in Eqn 4 and two traits; namely, WSG, the wood specific gravity, and LMA, the leaf mass per unit area (Wright *et* *al*., 2010; see Dataset S3). We fitted polynomial equations described as,

(Eqn 5) $$\begin{array}{cc} & B_{s}=5.57‐20.7{\text{WSG}}_{s}+14.99{\text{WSG}}_{s}^{2}‐0.004{\text{WSG}}_{s}{\text{LMA}}_{s}\\ & +0.09{\text{LMA}}_{s}‐0.0001{\text{LMA}}_{s}^{2}+\epsilon_{s,b} \end{array}$$

(Eqn 6) $$\begin{array}{ll} A_{s} & =‐2.36‐4.42B_{s}‐0.3B_{s}^{2}+0.12B_{s}{\text{LMA}}_{s}\\ & +0.08{\text{LMA}}_{s}‐0.001{\text{LMA}}_{s}^{2}+\epsilon_{s,a} \end{array}$$

($\epsilon_{s,a}$ and $\epsilon_{s,b}$, error terms).

As these fits explained a large proportion of variation in parameters A and B (see results), we sequentially used Eqns 5 and 6 to predict parameters B and A, respectively, for the 22 ERD species without direct data and estimated leaf hydraulic vulnerability curves using Eqn 4. We thus obtained parameters A and B for all of the 29 ERD species for use in Eqn 2. We also estimated species Ψ_20,leaf_ using their vulnerability curves.

#### Leaf area index (LAI)

In some of the alternative models for effective rooting depth, we explored the effect of seasonality in LAI on growth. We assumed species‐specific mean seasonal curves for LAI (standardized between 0 and 1; unitless), informed by a combination of long‐term records for leaf‐fall (Wright & Cornejo, 1990) and leaf lifetime (Osnas *et* *al*., 2018) (see Dataset S4; Methods S4; Fig S2).

#### Using ELM‐FATES to model soil matric potentials,

##### ELM‐FATES model description

We calibrated water availability by depth over the forest’s rooting zone, we used the Energy Exascale Earth System Land Model (ELM; Caldwell *et* *al*., 2019), coupled with the Functionally Assembled Terrestrial Ecosystem Simulator (FATES; Koven *et* *al*., 2020) (hereafter, ELM‐FATES). ELM is a land model that, among many features, simulates the physics and conservative dynamics of water, energy and carbon fluxes. In particular, soil hydrological fluxes are resolved vertically among discrete soil layers (1D) in a similar way to the CLM4.5 (Oleson *et* *al*., 2013). FATES is a community‐based, open‐source model used for studying climate–vegetation interactions. FATES is a vegetation demography model, with a size‐structured group of plants (cohorts) and successional trajectory‐based patches based on the ecosystem demography (Moorcroft *et* *al*., 2001) approach. FATES couples to ELM by a common interface of water and carbon fluxes. Detailed descriptions for ELM and FATES can be found elsewhere (Fisher *et* *al*., 2010, 2015; Bisht *et* *al*., 2018; Koven *et* *al*., 2020).

We ran ELM with FATES vegetation, in which the ELM model simulates interception, throughfall, canopy drip, infiltration, evaporation, surface runoff, subsurface drainage, redistribution within the soil column, and groundwater discharge and recharge so as to simulate changes in canopy water, surface water, soil water by depth and water in an unconfined aquifer (omitting processes relevant to snow, wetlands or lakes). (See Methods S5 for a water balance equation (Eqn S10) and a note on how soil water dynamics is simulated in ELM‐FATES.) The soil profile is discretized into ≤ 15 exponentially distributed soil layers with layer node depth z. Here, z ∈ Z; Z = (0.01, 0.03, 0.06, 0.12, 0.21, 0.37, 0.62, 1, 1.7, 2.9, 4.7, 7.8, 13) m.

#### ELM‐FATES model parameterization

In order to parameterize catchment hydrology in ELM‐FATES, we identified 11 parameters relevant for the water balance, determined their ranges based on literature for the study site, else for the tropics (Table S1), and ran 5000 simulations using Latin Hypercube Sampling (LHS; Stein, 1987) from this global parameter space. Notably, we leveraged local data for soil hydraulic conductivity by depth (Godsey *et* *al*., 2004; Fig. S3), and instead of the ELM default soil texture‐based pedo‐transfer functions, we estimated parameters of soil retention curves using existing data for gravimetric water content vs Ψ_soil_ (Kupers *et* *al*., 2019; Eqn S11). (See Methods S6.)

We ran ELM‐FATES for the 5000‐member 11‐parameter ensemble with hourly climate drivers measured at the BCI meteorological station over 1985–2018 (Faybishenko & Paton, 2021) initialized with the observed stand structure from the 50‐ha plot (Condit *et* *al*., 2019) in a single site mode. As our key interest here was on deriving soil water availability, we ran ELM‐FATES in a lower‐complexity configuration: static stand structure (see Methods S5), and with a single plant functional type (PFT) of evergreen trees. The latter was chosen as only 9.7% of BCI crown area is dry‐season deciduous (Condit *et* *al*., 2000) and addition of a dry‐deciduous PFT did not significantly alter results (not shown).

#### ELM‐FATES model calibration

We calibrated ELM‐FATES over 2012–2018 against three key fluxes and states in the water balance equation, namely: (1) evapotranspiration ET from the flux tower by the 50‐ha plot (2012–2017; Dataset S2; Table S2); (2) local stream discharge (2012–2018; Dataset S5; Paton, 2019b); and (3) soil volumetric water content (VWC) from two sources: (i) a long‐term (2012–2018) record of VWC averaged across three vertical time domain reflectometry (TDR) probes over the depth 0–15 cm from three locations near the flux tower (Dataset S6; Fig. S4), and (ii) plot‐wide snap‐shot measurements of VWC during the dry season of 2015 and 2016 at depths of 0.15, 0.4 and 1 m (1299 samples covering all soil types and habitats; Kupers *et* *al*., 2019). (See Methods S7.)

For ELM‐FATES calibration we calculated an objective function (Eqn S12) for each of the 5000‐member ensembles by equally weighting standardized root mean square error (RMSE) between observations and simulations across all fluxes and states mentioned above, and then identified 100 parameter ensemble members that minimized the objective function, ensuring that soil moisture dynamics‐by‐depth was captured correctly (Methods S7). (See Table S1 for the ranges of best‐fit values for different parameters.)

#### Soil water potential dynamics and hydrological droughts

We ran ELM‐FATES with the best‐fit 100 ensemble members from 1985–2018, with the first five years used for model spin‐up. Extreme hydrological droughts were identified by depth z as days for which $\Psi_{\text{soil},z}$ was more negative than the 5^th^ percentile of $\Psi_{\text{soil},z}$ for a given day of the year.

#### ELM‐FATES model evaluation

We evaluated ELM‐FATES by calculating RMSE between simulated and observed long‐term daily VWC for the depths of 0.1, 0.4 and 1 m (2016–2018), based on a dataset we had left out during calibration. We obtained these observations from three horizontal TDR probes at the depths of 0.1, 0.4 and 1 m at a location near the vertical probes (Dataset S6).

#### ERD model structure selection

We used δ ^2^H_xylem_ as an independent observation to validate the ERD models. As root water‐uptake is generally a nonfractionating process, tree δ ^2^H_xylem_ reflects the signature of source water. Given a vertical gradient of δ ^2^H in soil and groundwater, δ ^2^H_xylem_ provides an index of rooting depth (Dawson & Ehleringer, 1991). We leveraged δ ^2^H_xylem_ from BCI (Meinzer *et* *al*., 2001) for the dry season of March 1997 as this period showed largest seasonal divergence in δ ^2^H_xylem_ among species and vertically in soil and groundwater δ ^2^H at natural abundance level (Fig. S5).

For comparison with modeled ERD, we removed six species from the Meinzer *et* *al*. (1999) dataset to account for the uncertainty in their water‐sourcing depths. δ ^2^H_xylem_ from leafless trees may not be linked with water sourced at the time of measurement and thus may not be comparable to species that had leaves. Leaflessness status of sampled trees is not recorded in Meinzer *et* *al*. (2001). We therefore removed five species that are typically leafless in March–April (Joseph S. J. Wright, personal observation), that is, the months of δ ^2^H_xylem_ sampling. δ ^2^H to soil depth relationship was particularly uncertain for δ ^2^H > −40‰, so from the remaining dataset, we removed one species, *Guapira* *standleyana*, with δ ^2^H_xylem_ of −28.9‰ ± 3.7SE (see Fig. S5). For each model of ERD (Eqns 3, S1–S6), modeled species ERD was regressed against species δ ^2^H_xylem_ for a maximum of six species.

### Relationships between ERD and aboveground hydraulic traits

We evaluated whether ERD was associated with aboveground hydraulic traits sourcing the latter from existing datasets (Wolfe *et al*., 2019, 2021) for seven species that overlapped with our assessment of ERD. We regressed species ERD against maximum stem hydraulic conductivity $K_{\text{max,stem}}$ (*n* = 7), leaf turgor loss point Ψ_tlp_ (*n* = 7), vulnerability to embolism from cavitation Ψ_88,stem_ measured in terms of pressure at which 88% of $K_{\text{max,stem}}$ is lost (*n* = 7), and hydraulic safety margins Ψ_min_ – Ψ_88,stem_ (*n* = 6). (See Datasets S7–S9 and Table S3 for data collection and estimation of these variables.)

### Mortality analyses and species‐specific drought exposure

In order to test whether ERD plays a role in mitigating mortality risk, we calculated mortality rates for large trees (here, ≥ 10 cm dbh) in the 50‐ha plot for species with ERD estimates (all canopy species with maximum height ≥ 30 m) as well as average abundance of ≥ 20 trees in the plot (n = 28) (Condit *et* *al*., 2019). For each of the seven census intervals t in the 35‐yr record (1981, 1985, 1990, 1995, 2000, 2005, 2010, 2015), mortality rate, $M_{t}$ (% yr^−1^) for species s for two successive censuses, $c_{1}$ and $c_{2}$, was calculated as $M_{s,t}=\frac{D_{s,c_{2}}}{N_{s,c_{1}}}\times \frac{100}{d}$, where $N_{s,c_{1}}$ and $D_{s,c_{2}}$ are the total number of large trees of species s present in the 50‐ha plot in $c_{1}$ and dead in $c_{2}$, respectively, and d is the duration based on mean dates of $c_{1}$ and $c_{2}$.

Dry season deciduous species may escape drought exposure via leaf deciduousness, so we analyzed deciduous and evergreen species separately. Tree species on BCI are scored by expert botanists as one among four leaf habits – evergreen, brevideciduous, facultative deciduous and obligate deciduous (Meakem *et* *al*., 2018; Dataset S4). We pooled all deciduous leaf habits together (hereafter, ‘deciduous’ for brevity) and regressed species ERD against species $M_{s,t}$ for each census interval t for deciduous (n = 16) and evergreen species (n = 12) separately.

As an indicator of exposure to water stress, we use a species‐specific critical hydraulic threshold, Ψ_crit_, here defined as Ψ_20,leaf_. The duration of exposure to water stress, and thus the potential for realized hydraulic risk, was defined for each species as the proportion of days in each census interval t of the $\Psi_{\text{soil}}$ simulation period (1990–2015) during which soil water potentials in the soil layer matching species ERD, $\Psi_{\text{soil},z=\text{ERD}}$, were more negative than species Ψ_crit_.

All statistical analyses were conducted in the R statistical environment (v.4.0.3; R Core Team, 2020).

## Results

### Soil water dynamics by depth and hydrological droughts

**Fig. 2:**
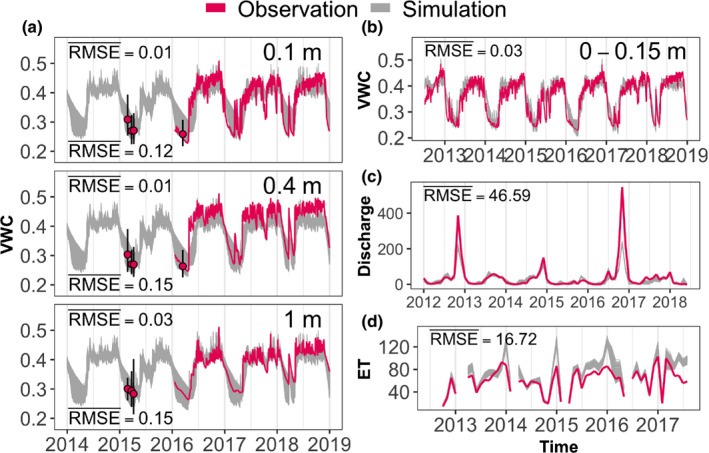
Energy Exascale Earth System Land Model coupled with the Functionally Assembled Terrestrial Ecosystem Simulator (ELM‐FATES) calibration and evaluation. Observations (red lines and points) vs simulations (gray lines) from 100 best‐fit ensemble‐member runs of ELM‐FATES are shown for daily volumetric water content (VWC) by depth for horizontal time domain reflectometry (TDR) probes at three depths (0.1, 0.4 and 1 m; a) and for an average of three vertical TDR probes (0–0.15 m; b); monthly stream discharge (Discharge, c) and monthly evapotranspiration from the flux tower (ET, d). Red points in panel a show average, manual plot‐wide observations of VWC with 95% CI (error bars). All observations (red lines and points) were in‐sample, except for VWC data from the horizontal probes (red lines in a) which were out‐of‐sample. Values in inset are average RMSE across the 100 best‐fit simulations. For (a), values at the top are for manual, plot‐wide VWC and those at the bottom are for VWC from TDR probes. VWC is in units of cm^3^ cm^−3^, whereas ET and Discharge are in mm per month.

The 100 ELM‐FATES ensemble members with the best fits to observed soil moisture dynamics captured soil moisture seasonality at multiple depths (Fig. 2a,b), including the out‐of‐sample observations. These simulations also captured the dynamics in stream discharge (Fig. 2c) and in evapotranspiration (Fig. 2d), slightly underestimating peak discharge in wet years and slightly overestimating peak ET. The reduction in parameter range (Table S1) in the best‐fit ensembles compared to the tested global ranges showed that the model calibration was primarily sensitive to the Ball‐Berry stomatal slope parameter (fates_leaf_BB_slope), the ELM root distribution parameter that regulates the depth of the rooting profile (fates_rootb_par), soil hydraulic conductivity (HKSAT) profile especially at depth, and the adjustment factor (HKSAT_ADJ) that modifies soil hydraulic conductivity to account for macroporosity and direct flow paths (Figs S6, S7). The distribution of maximum depth of soil water dynamics, and thus ecosystem root zone depth, across the 100 hydrological realizations encompassed 95% CI of 2.9–13 m (median 4.7 m).

**Fig. 3:**
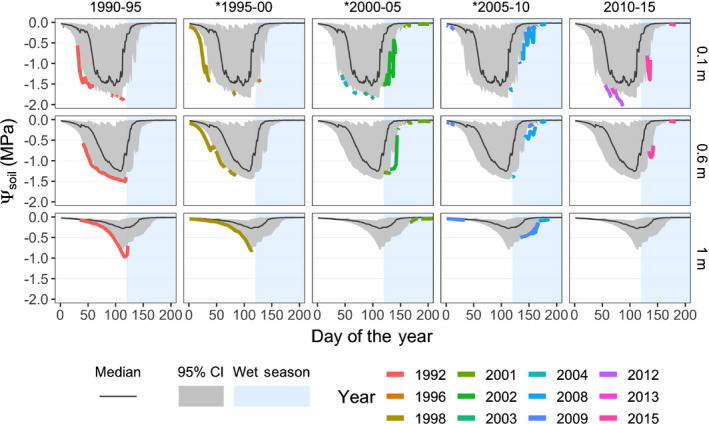
Occurrence of extreme hydrological droughts by census intervals (horizontal panels) at three representative depths: 0.1, 0.6 and 1 m (vertical panels). Mean (black lines) and lower half of 95% distribution (grey areas) of soil water potential $\Psi_{\text{soil},z}$ for a given day of year (DOY) and depth z are shown (same across all census intervals). $\Psi_{\text{soil},z}$ by DOY for a year (colored line) is only shown if at least one DOY $\Psi_{\text{soil},z}$ was more negative than the 5^th^ percentile of $\Psi_{\text{soil},z}$ for that DOY. Note the distinctive features of extreme droughts that occurred during the three periods indicated by asterisks: all three periods featured extreme hydrological droughts that either prolonged over the dry season (1995–2000), or occurred in the wet season, effectively extending the dry season (2000–2005, 2005–2010).

Analysis of soil water potential dynamics $\Psi_{\text{soil},z}$ by depth *z* from 0.01 to 13 m obtained from the best‐fit simulations (Fig. S8) revealed that every 5‐yr census interval had at least one extreme hydrological drought year, but each interval varied in terms of number of extreme years, drought intensity, drought seasonality and duration (Fig. 3). Hydrological droughts in census interval 1990–1995 were marked by a prolonged dry season, whereas those during 2000–2005 and 2005–2010 distinctly occurred in the wet season, effectively extending the dry season into the wet season (Fig. 3). Across the simulation period 1990–2015, simulated $\Psi_{\text{soil},z}$ remained above −0.5 MPa from depths of 1.7–13 m (Fig. S8).

### Predictors of leaf hydraulic vulnerability curves

**Fig. 4:**
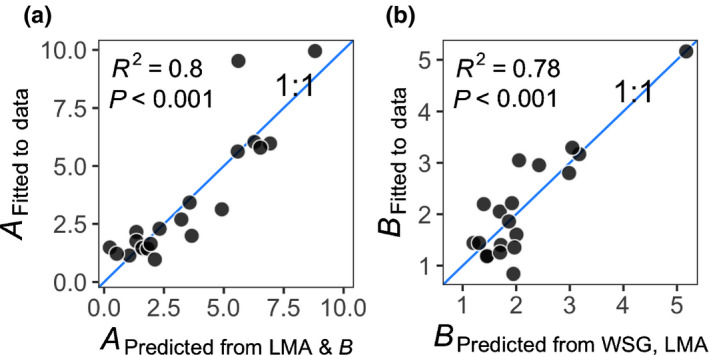
Parameters A (panel a) and B (panel b) for the leaf vulnerability curves that were fitted to observed data on $K_{\text{leaf}}$ vs Ψ_leaf_ for 21 species (Eqn 4) vs those that were predicted from a set of models based on trait‐proxies (Eqns 5, 6). Goodness‐of‐fit (R ^2^) and significance levels are given in inset.

Parameters of leaf vulnerability curves were predictable from WSG and LMA (Fig. 4). WSG and LMA explained a large proportion of variance in parameter B (Eqn 5; Adj. R ^2^ = 0.69, *P* < 0.001). WSG and parameter B explained a large proportion of variance in parameter A (Eqn 6; Adj. *R* ^2^ = 0.74, *P* < 0.001; see also Fig. S9). This predictive power allowed us to estimate leaf vulnerability curves used in the ERD models for 22 ERD species that lacked direct observations (among 29 ERD species) (Table S4; Fig. S10).

### Effective rooting depths

**Fig. 5:**
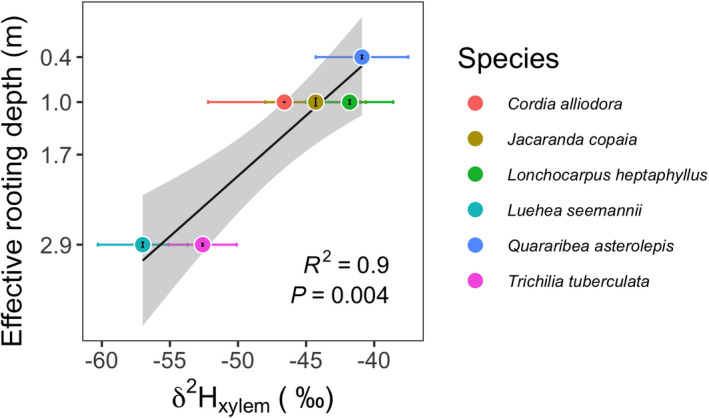
Modeled effective rooting depth (ERD; mean ± 1SE (m)) vs dry‐season stable isotopic concentration δ ^2^H_xylem_ (mean ± 1SE; ‰) for six canopy species from Barro Colorado Island. δ ^2^H_xylem_ data are from Meinzer *et* *al*. (1999).

The best ERD model (Eqn 3; Notes S1) explained a large fraction of the variance in δ ^2^H_xylem_ (R ^2^ = 0.9, *P* = 0.004, n = 6; Fig. 5; see also Fig. S11). This model included an effect of VPD and not LAI (Eqn 3).

**Fig. 6:**
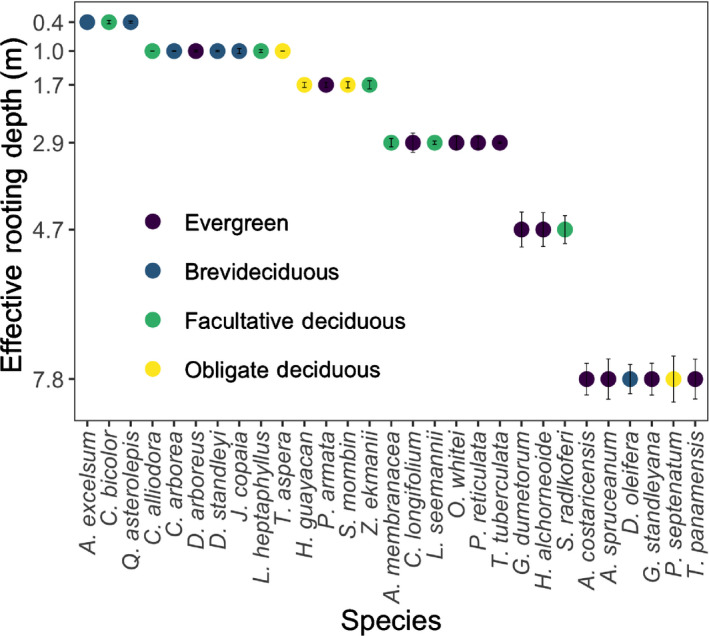
Modeled effective rooting depth (ERD; mean ± 1SE (m)) for 29 large, canopy species of Barro Colorado Island. A species ERD is defined as the median, across 100 hydrological realizations, of soil layer depth z at which soil water dynamics ($\Psi_{\text{soil,}z}$) best explained observed dynamics of species growth for each realization. As soil layers in the hydrological model are discretely resolved into exponentially increasing depths, so is the ERD axis. Species are color coded by leaf habit. See Table S4 for species' complete scientific names.

Modeled ERD for the 29 large (≥ 30 cm DBH) trees of canopy species varied from 0.4 m to 7.8 m (Fig. 6). Evergreen and deciduous species had similar ranges of ERDs, but a greater proportion of deeper ERD species tended to be evergreen rather than deciduous – a group composed of a variety of categories of deciduousness (Fig. 6). Notably, two species, *Luehea* *seemannii* and *Trichilia* *tuberculata*, that Meinzer *et* *al*. (1999) found to have δ ^2^H_xylem_ values between soil water and groundwater, suggesting that these species sourced most of the water from depths > 1 m and likely to have sourced some portion of groundwater, also were identified by our model with ERD > 1 m (2.9 m for both species; Fig. 5).

### Relationships between ERD and aboveground hydraulic traits

**Fig. 7:**
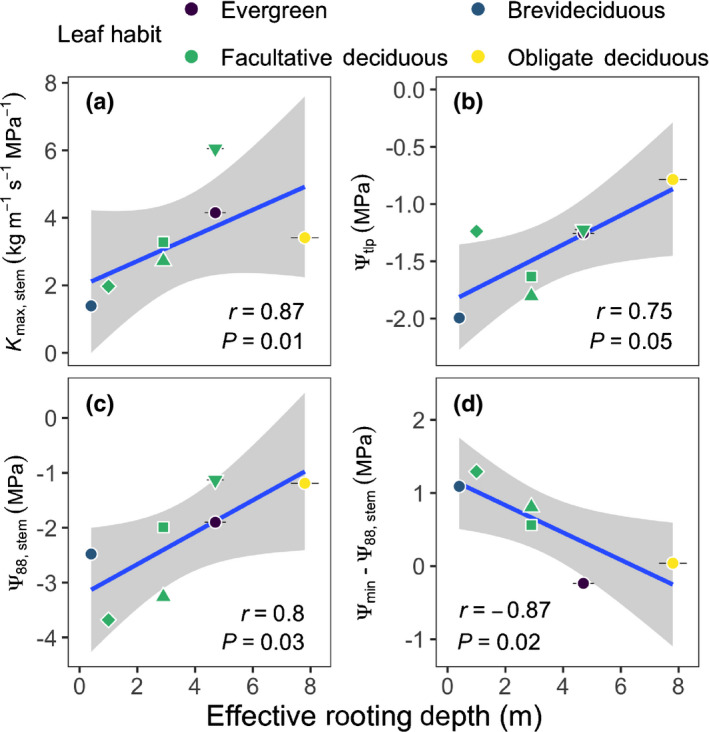
Modeled effective rooting depth (ERD) vs hydraulic properties for seven canopy species found on Barro Colorado Island (Panama); namely, maximum stem area‐specific hydraulic conductivity of stem ($K_{\text{max,stem}}$; a), bulk leaf turgor loss point, the Ψ_leaf_ where turgor potential = 0 (Ψ_tlp_; b), Ψ_stem_ at 88% loss of stem conductivity (Ψ_88,stem_; c), and aboveground hydraulic safety margin (Ψ_min_–Ψ_88,stem_; d). Spearman’s r and significance levels are given in panel insets. Linear model fits (blue lines) with confidence bands (gray area) are shown for significant fits at α = 0.05. Species are color‐coded by leaf habit. Multiple species in Facultative deciduous leaf‐habit are distinguished by shapes.

Species with deeper ERD showed greater $K_{\text{max,stem}}$ (Spearman’s r = 0.87, *P* = 0.01; Fig. 7a), less negative leaf Ψ_tlp_ (r = 0.75, *P* = 0.05; Fig. 7b), less negative Ψ_88,stem_ and thus greater vulnerability to xylem embolism from cavitation (r = 0.8, *P* = 0.03; Fig. 7c), and narrower aboveground hydraulic safety margins (Ψ_min_ – Ψ_88,stem_, r = −0.87, *P* = 0.02; Fig. 7d). (See Fig. S12 for the full correlation matrix.)

### Effective rooting depths, mortality and hydrological droughts

**Fig. 8:**
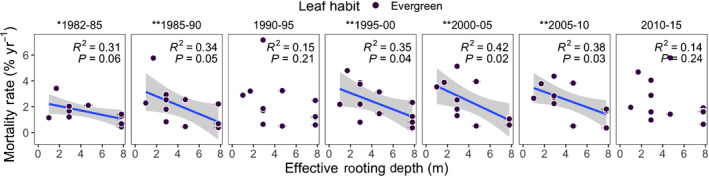
Mortality rate (mean ± 1SE (% yr^−1^)) vs modeled effective rooting depth (ERD; mean ± 1SE (m)) for 12 evergreen, canopy species found on Barro Colorado Island over seven census intervals (1981–2015). R ^2^ and significance levels for linear model fits are given in panel insets. Model fits (blue lines) with confidence bands (gray area) are only shown for periods with significant fits. Census interval significance: **, α = 0.05; *,α = 0.1.

Among the seven census‐intervals over 1982–2015 for which we analyzed relationship of ERD with mortality rates, six intervals were associated with occurrence of one or more El Niño events (Condit, 2017; Detto *et* *al*., 2018). Of the six intervals with El Niño events, ERD explained 30–40% variation in mortality rates among evergreen species during five intervals (1982–1985, 1985–1990, 1995–2000, 2000–2005 and 2005–2010) such that species mortality rates decreased with deeper ERD (*P*‐values < 0.05 for four intervals and 0.06 for one interval; Fig. 8). For deciduous species, ERD explained 11–16% variation in mortality in four intervals with El Niño events, but *P*‐values were not significant (Fig. S13).

Our analysis of hydrological droughts ranged from 1990 to 2015 and revealed distinctive extreme, prolonged hydrological droughts for the census‐intervals 1995–2000, 2000–2005 and 2005–2010, for which ERD explained significant mortality (Fig. 3). ERD also explained significant mortality in earlier droughts (1982–1985 and 1985–1990; Condit, 2017), not covered by our $\Psi_{\text{soil}}$ estimates.

**Fig. 9:**
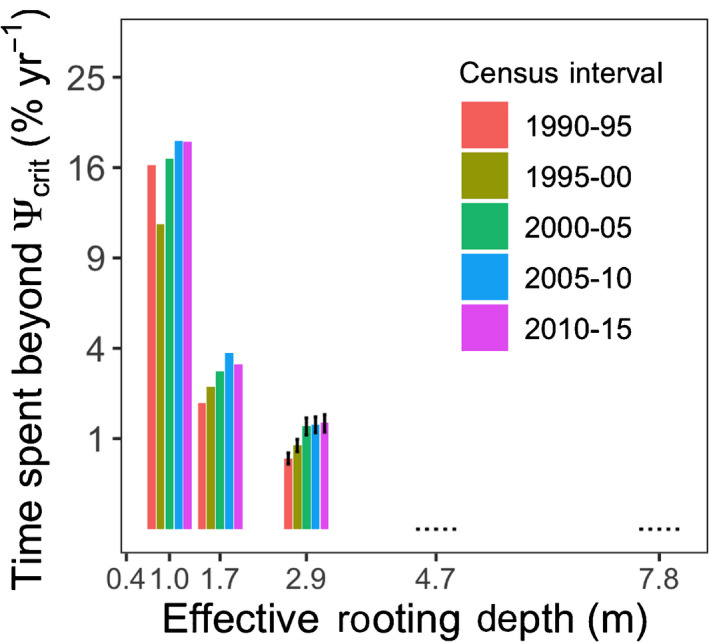
Modeled effective rooting depth (ERD; horizontal‐axis) vs time spent beyond critical hydraulic threshold (vertical axis) by census interval (colored bars) for 12 evergreen species included in the mortality analyses (Fig. 8). Each bar represents the average time species of the same ERD spent beyond species‐specific critical hydraulic thresholds in a given interval, that is, the proportion of days for which $\Psi_{\text{soil},z=\text{ERD}}$ was more negative than species Ψ_crit_, defined as Ψ_20,leaf_, and where z is the soil depth matching species ERD. SEM shown over each bar when available. Note the squared *y*‐axis scale.

On average, species exposure to water stress (% days $\Psi_{\text{soil},z=\text{ERD}}$ < Ψ_crit_) exponentially declined with ERD (Figs 9, S14), indicating that species with shallower ERD spent greater time under significant hydrological drought, and thus likely experienced greater hydraulic risk (Notes S2). Exposure to water stress increased over the three periods for which ERD explained significant mortality (Figs 9, S11), although it also was high in 1990–1995 for the shallowest ERD (Fig. 9), but without elevated mortality rates (Fig. 8).

## Discussion

We introduce a novel approach for estimating effective rooting depths (ERD) using 25 years of tree growth, species‐specific leaf vulnerability curves, modeled soil water potential profiles, and observed vapor pressure deficit (VPD) (Eqn 3; Figs 1, 6). Our predictions of ERD were consistent with estimates using δ ^2^H_xylem_ (Fig. 5). Our analyses suggest that co‐occurring large canopy tree species with deeper ERD were associated with higher aboveground hydraulic efficiency, but lower safety (Fig. 7). Nevertheless, evergreen but not deciduous species with deeper ERD showed significantly lower mortality rates than shallower ERD species (Figs 8, S11). This ERD–mortality relationship was significant in five of six census intervals that had experienced one or more El‐Niño events, over seven census intervals studied in total (1982–2015). Quantifying extreme droughts in the whole soil column over 1990–2015 revealed that ERD explained significant levels of mortality in periods when extreme soil droughts prolonged the dry season water stress (Fig. 3). Species exposure to water stress exponentially declined with deeper ERD (Fig. 9). Because extreme, sustained water stress increases risk of mortality via hydraulic failure and/or carbon starvation, deeper ERD may mitigate drought‐induced mortality by limiting exposure to water stress (Rowland *et* *al*., 2015; McDowell *et* *al*., 2018b). We thus demonstrate, for the first time, a link between species trade‐offs in above‐ and belowground hydraulic traits, drought exposure through extreme hydrological droughts quantified over the whole soil column, and large (≥ 10 cm diameter at breast height) tree mortality across several El‐Niño events over 35 years.

### Drought strategies designed to mitigate realized hydraulic risks

If investments in stress‐tolerance traits come at a cost, such insurance may not pay off if the risk of stress is not realized. Our findings suggest, on the one hand, that species with investment in deep roots can afford the hydraulically efficient, but risky, suite of traits (Fig. 7), because access to a reliable deep‐water resource ensures that for them hydraulic risk is not realized (Fig. 9). On the other, shallow‐rooted species pay the cost of hydraulic safety in terms of efficiency, adapted for an environment in which hydraulic risk is significant, as extreme droughts cause exponentially greater water stress in shallow soil layers (Figs 9, S6).

Extreme, prolonged El‐Niño droughts (Fig. 3) in our study may have crossed even the greater tolerance limits of shallow‐rooted species leading to their greater mortality compared to deep‐rooted species (Fig. 8). Hydraulic risk for shallow‐rooted species may have been exacerbated in our 35‐yr study period in which extreme water‐deficit years were more frequent than those in the last century (Condit, 2017). The greater survival of deep‐rooted species that we observed may not continue into the future if droughts intensify.

We found significant ERD–mortality relationships during extreme water stress for evergreen but not deciduous species (Figs 8, S11); consistent with the expectation that deciduous species also can avoid water stress via leaf drop. This also is consistent with the observation that species distributions along local (BCI) and regional (Panama) moisture gradients are correlated with leaf turgor loss point (Ψ_tlp_) for evergreen but not deciduous species (Kunert *et* *al*., 2021). Studies that simultaneously assess coordination between leaf phenology, rooting depth and hydraulic traits are almost absent for the tropics and warrant future consideration (Oliveira *et* *al*., 2021). Our analyses for ERD–hydraulic trait relationships were limited to only seven species, but covered all species of different leaf phenologies (Fig. 7), so here we assume that the trend in the ERD–hydraulic trait relationships holds true across all species of different leaf phenologies. We also found that the range of ERDs overlapped across leaf phenology, but evergreen species tend to have deeper ERDs (Fig. 6), consistent with observations by Meinzer *et* *al*. (1999) and elsewhere (Fan *et* *al*., 2017; Smith‐Martin *et al*., 2020; Oliveira *et* *al*., 2021). Whether deciduous species also have more efficient and vulnerable hydraulics at BCI as is observed elsewhere remains to be studied (Markesteijn *et* *al*., 2010, 2011; Gleason *et* *al*., 2016; Xu *et* *al*., 2016). Leaf phenologies at BCI are numerous and complicated and warrant further research.

### Drought exposure integral to assessing drought‐induced mortality

Our study brings attention to the need for assessing drought sensitivity in terms of species drought exposure and realized hydraulic risk by accounting for hydrological drought and tree rooting depths. We found that species accessing deeper water had greater xylem vulnerability to embolism and narrower branch hydraulic safety margins (Fig. 7). These traits are commonly identified as proxies for mortality risk (Anderegg *et* *al*., 2016), but in fact were associated with species with less drought exposure that had lower mortality. Hydraulic risk was balanced by investment in deep roots (Figs 3, 8).

Our results are consistent with recent studies that analyzed rooting or water‐sourcing depths vs hydraulic traits and mortality rates during extreme droughts (Nardini *et* *al*., 2015; Venturas *et* *al*., 2016; Johnson *et* *al*., 2018; see also Brum *et* *al*., 2017, 2019; Rowland *et* *al*., 2015). Globally, large trees tend to exhibit greater growth reductions, lower post‐drought resilience and greater increases in mortality relative to their understory counterparts (Phillips *et* *al*., 2010; Bennett *et* *al*., 2015). Our finding that deep‐water access buffers drought‐induced mortality in large trees is relevant for understanding drought resistance, resilience and recovery (Bennett *et* *al*., 2015; McGregor *et* *al*., 2021). Future studies should test the ERD–mortality relationship on a greater number of species.

Our result of lower mortality in deep‐rooted trees contrasts with the inverse model finding of Chitra‐Tarak *et* *al*. (2018) (hereafter, CT2018) in which deeper ERD species in a South‐Asian seasonally dry tropical forest had higher mortality in a rare, prolonged drought. The hydrological model in CT2018 revealed that the multi‐year drought exhausted the deep soil and even bedrock water availability (also see, Goulden & Bales 2019; Ivanov *et* *al*., 2012). By contrast, our hydrological modeling at BCI found that Ψ_soil_ for depths deeper than 2.9 m did not cross critical hydraulic threshold (Ψ_crit_) of the most sensitive tree species (−0.17 MPa) for all of the dry seasons and droughts during 1990–2015 (Notes S1). Mean annual rainfall of 1095 mm in CT2018 compared to 2627 mm at BCI, and precipitation to potential evapotranspiration ratio of nearly one in CT2018 compared to nearly two in BCI, are major factors in the different mortality responses. At BCI, deep soil layers were recharged annually (Fig. S6), whereas in CT2018 they were not. The contrast between the two studies highlights the combined role of seasonal precipitation input and site‐specific hydrology in modulating the mortality risk for deep‐rooted species.

### Modeling effective rooting depths at the tree community level

By estimating ERD for large trees of 29 canopy tree species (Fig. 6), we make an important advance in modeling effective rooting depths at the community level in species‐rich tropical forests. Our best ERD model is a key improvement over the model of CT2018 as we employ a physiologically meaningful representation, fewer parameters and corroboration with tree and soil data for δ ^2^H.

Our ERD model predicts daily maximum diurnal leaf hydraulic vulnerability (Ψ_leaf_) assuming that it is equivalent to Ψ_soil_. Our model ignores other factors that buffer soil drying such as stem water storage capacitance (Wolfe, 2017), and may thus have overestimated ERD, especially in those species for which capacitance is important as a drought‐avoidance strategy, for example, the deciduous species (Borchert & Pockman, 2005). Future studies should investigate the role of capacitance on estimating ERD.

Although ERD models have the potential to estimate ERDs for entire tree communities, during their development phase, ERD models may need to be validated against data for a subset of representative species of the community, as this study did. ERD models should be tested across varied climates and forest types, covering contrasting plant strategies and possibly seasonality in ERDs. Direct observations of rooting depths and stable water isotope‐based water‐sourcing depths will be important datasets for such validation; although the interpretation of isotopic data is still under research (Adams *et* *al*., 2020; Bowers *et* *al*., 2020; Deurwaerder *et* *al*., 2020).

### Future directions

The relationships that we identify between above‐ and belowground traits, vertical profile of soil water status and mortality rates are important for representing diversity in dynamic global vegetation models (DGVMs), which intrinsically rely on the parameterization of contrasting life history strategies (Scheiter *et* *al*., 2013) and the simulation of competition between those strategies. In the context of trait filtering models, if we used the hydraulic trait information without knowledge of their relationship to rooting depth, models would likely kill the ‘risky’ strategy trees in droughts, which would, in fact, be the opposite result from that observed in this study. A key outcome of this study is thus the relationships between hydraulic traits and ERD that could be plugged into a DGVM of BCI. To assimilate ERDs, DGVMs could vary rooting parameters such that the centroid of the species water‐uptake profiles match ERDs. We found that leaf mass per unit area (LMA) and wood specific gravity (WSG) were strong predictors of leaf hydraulic vulnerability curves (Fig. 4). Albeit future studies should undertake sensitivity analyses for uncertainties involved, our finding offers the promise of a greater ability to parameterize the ‘hard’ hydraulic traits with the abundant ‘soft’ trait data, thus allowing for a better representation of forest hydrodynamics. The relationships between ERD and aboveground hydraulic traits that we find, thus provide important insights on how to model rooting depths and their coordination or trade‐offs with other traits, in order to better represent the functional diversity of tropical forests and their trajectories into the future.

Our inverse ERD model was parameterized on 5‐year growth data, with five data points over a 25‐yr period, which decoupled climate events and demographic outcomes. Future studies could better constrain the ERD model with higher frequency growth data such as those from dendrometer bands. At high temporal resolution, however, the role of reversible dehydration in tree diameter change increases (Chitra‐Tarak *et* *al*., 2015; Chitra‐Tarak, 2016; Mencuccini *et* *al*., 2017), but that may provide an avenue to include stem water storage and dynamic rooting depths in ERD models. Three of our exploratory ERD models included leaf area index (LAI) seasonality, but we did not select them as they worsened the fit with growth data for many species (Fig. S9). Our interpretation of this result is that VPD and leaf hydraulic vulnerability curves may be adequate to explain inter‐census differences in growth (via stomatal control), but also acknowledge that our estimates of seasonality of LAI is a tentative estimate that combines leaf‐fall data and the timing of leaf‐gain backtracked from leaf lifetime, and omits inter‐annual variation. Species‐level leaf‐fall data from litter‐traps that we used includes large within‐species variability in leaf‐fall timing, and so may not have captured tree‐level seasonality in deciduousness (Methods S4). Future studies may improve models of LAI seasonality and make use of data from new technologies such as drone based monitoring of LAI in species‐rich tropical forests (Park *et* *al*., 2019).

Although our ERD model empirically predicts growth via estimating Ψ_leaf_ from Ψ_soil_ of a specific depth, mechanistic models that account for plant hydrodynamics and other processes influencing growth are likely to predict growth more accurately, and thus ERD and hydraulic risk (Sperry *et* *al*., 1998; Christoffersen *et* *al*., 2016; Duursma *et* *al*., 2018; Yang *et* *al*., 2019). Although data needs for parameterizing such models could be greater (e.g. hydraulic vulnerability curves and capacitance for roots, stems and leaves), adding a degree of parameter uncertainty in community‐wide application, such models hold greater promise in improving our understanding of plant physiology (see, e.g., Johnson *et* *al*., 2018).

As in CT2018, with water availability resolved for a 1D column, we interpret our ERD estimates as revealing relative differences among species’ effective rooting depths rather than absolute depths. To estimate absolute depths and topographic variation in ERD within or across species, future studies may use soil moisture dynamics from a distributed hydrological model (e.g. Schwantes *et* *al*., 2018). We note that data availability on soil water retention curves (Ψ_soil_ vs volumetric water content (VWC)), hydraulic conductivity (*K*_soil_) by depth, soil moisture by depth, stream discharge and evapotranspiration were important for effective calibration of our 1D hydrological model. We recommend widespread and coordinated collection of these variables as well as water‐table levels in forest‐inventory sites to allow for estimation of tree water environments.

### Conclusions

Establishing relationships between environment, traits and demographic outcomes of plants is imperative for developing a predictive plant ecology. Tree rooting depths and actual water environments through hydrological rather than meteorological droughts nonetheless are rarely studied. To the best of our knowledge, this is the first study to test for a mechanistic link between plant‐available water in the whole‐soil column, tree above‐ and belowground hydraulic architecture and long‐term mortality outcomes for a species‐rich forest. We report here that deep‐water access plays a role in mitigating mortality of otherwise vulnerable stem hydraulics. This has important implications for our predictive understanding of tropical forest dynamics under current and future climate. Our community‐scale framework for modeling effective rooting depths and leaf vulnerability curves indicates the possibilities in expanding the use of these critical, rare observations in species‐rich forests towards community‐scale generalizations.

## Author contributions

Conceptualization: RC‐T. Data curation: BF, SRP for climate drivers. SRP for stream discharge. MD for GPP, ET, TDR, bulk density. SJK for manual GWC and GWC vs $\Psi_{\text{soil}}$. NK, JZ, KJA‐T, LS for leaf hydraulics, BTW for stem hydraulics. RP and SA for the BCI 50ha plot censuses. SJW for maximum tree height, WSG, LMA, deciduousness, leaf fall and leaf lifetime. Formal analysis: RC‐T, CX. MD for upgrading GPP‐VPD relationship and LAI seasonality. Funding acquisition: SMM, JC, CX, NGM, LMK. Investigation: RC‐T, CX (SMM and LR for an exploratory version). Methodology: RC‐T, CX (SMM and LR for an exploratory version). Project administration: RC‐T, Resources: SMM, BF, SRP, MD, SJK, NK, JZ, KJA, LS, BTW, SJW. Software: RC‐T; RAF, RGK, CDK, CX, RC‐T for ELM‐FATES. Supervision: CX, SMM, BDN, Validation: RC‐T. Visualization: RC‐T, Writing – original draft: RC‐T. Writing – review & editing: RC‐T, CX, KAT, MD, RAF, RGK, CDK, LMK, NK, SJK, NGM, BDN, SRP, LR, LS, JMW, BTW, CW, SJW, JZ, SMM. See https://casrai.org/credit/ for the taxonomy of credits.

## Supporting information

**Dataset S1** Microclimatic and flux tower data.
**Dataset S2** Leaf hydraulic conductivity and vulnerability to cavitation.
**Dataset S3** Wood specific gravity (WSG) and leaf mass area (LMA).
**Dataset S4** Leaf deciduousness categories.
**Dataset S5** Stream discharge from the Conrad catchment.
**Dataset S6** Volumetric water content.
**Dataset S7** Stem maximum hydraulic conductivity and vulnerability to cavitation.
**Dataset S8** Leaf turgor loss point.
**Dataset S9** Aboveground hydraulic safety margins.
**Fig. S1** Observed relationship between GPP and VPD at Barro Colorado Island.
**Fig. S2** Seasonal variation in estimated standardized LAI for 29 species at Barro Colorado Island.
**Fig. S3** Ensembles of soil hydraulic conductivity variation by depth for Conrad Trail Stream Catchment, Barro Colorado Island (data from Godsey *et* *al*., 2004).
**Fig. S4** Volumetric water content vs. TDR data for a vertical TDR probe used for TDR probe calibration.
**Fig. S5** δ^2^H of soil water by depth measured at Barro Colorado Island (data from Meinzer *et* *al*., 1999).
**Fig. S6** Parameter sensitivity of ELM‐FATES soil water content and evapo‐transpiration.
**Fig. S7** Parameter sensitivity of ELM‐FATES stream discharge.
**Fig. S8** ELM‐FATES predicted soil water potential dynamics by depth over 1990–2018.
**Fig. S9** Leaf hydraulic vulnerability curves fitted to observed *K*_leaf_ vs Ψ_leaf_ data for 21 tree species from Barro Colorado Island.
**Fig. S10** Percent loss of leaf hydraulic conductivity curves for ERD species, including curves fitted to *K*_leaf_ vs Ψ_leaf_ as well as those based on scaling relationships with LMA and WSG.
**Fig. S11** Effective rooting depths (ERD) vs δ^2^H_xylem_ for each of the ERD model structures tested.
**Fig. S12** Correlation matrix between ERD and hydraulic traits.
**Fig. S13** Mortality rates for deciduous species across census intervals from 1985–2015 vs ERD.
**Fig. S14** Modeled effective rooting depths of deciduous species vs time spent beyond critical hydraulic threshold.
**Methods S1** Alternative structures for effective rooting depth model.
**Methods S2** Statistics for identifying best‐fit ERD.
**Methods S3** Processing of forest census data for growth estimates.
**Methods S4** Leaf area index calculations.
**Methods S5** Details of the ELM‐FATES model.
**Methods S6** Details for ELM‐FATES model parameterization.
**Methods S7** ELM‐FATES calibration.
**Notes S1** ERD model structure selection.
**Notes S2** Additional results for exposure to water stress.
**Table S1** ELM‐FATES parameters used to generate ensembles, with description, prescribed global ranges, rationale for the choice of ranges and references, as well as ranges for best‐fit ensembles.
**Table S2** QA/QC procedure applied to eddy covariance fluxes.
**Table S3** Aboveground hydraulic traits data from Wolfe *et* *al*. (2019) and Wolfe *et* *al*. (2021) used for comparison with ERD.
**Table S4** Leaf vulnerability curve parameters A & B, *K*_max, leaf_ and Ψ_20,leaf_ by source (data or model) for the 29 species with ERD estimates.
Please note: Wiley Blackwell are not responsible for the content or functionality of any Supporting Information supplied by the authors. Any queries (other than missing material) should be directed to the *New* *Phytologist* Central Office.

## Data Availability

ELM‐FATES source code, simulation outputs for best‐fit parameter ensemble members and all of the R scripts to reproduce the manuscript are available (Chitra‐Tarak *et al*., 2020). Data sources for all other datasets used are provided throughout the manuscript.
