# Supplementary material for: Hydraulically‐vulnerable trees survive on deep‐water access during droughts in a tropical forest
Source: New Phytol. 2021 Jul 2;231(5):1798–813. doi: 10.1111/nph.17464 (PMC8457149; doi:10.1111/nph.17464)
Supplement: Supplementary file 1 — Dataset S1 Microclimatic and flux tower data. Dataset S2 Leaf hydraulic conductivity and vulnerability to cavitation. Dataset S3 Wood specific gravity (WSG) and leaf mass area (LMA). Dataset S4 Leaf deciduousness categories. Dataset S5 Stream discharge from the Conrad catchment. Dataset S6 Volumetric water content. Dataset S7 Stem maximum hydraulic conductivity and vulnerability to cavitation. Dataset S8 Leaf turgor loss point. Dataset S9 Aboveground hydraulic safety margins. Fig. S1 Observed relationship between GPP and VPD at Barro Colorado Island. Fig. S2 Seasonal variation in estimated standardized LAI for 29 species at Barro Colorado Island. Fig. S3 Ensembles of soil hydraulic conductivity variation by depth for Conrad Trail Stream Catchment, Barro Colorado Island (data from Godsey et al., 2004). Fig. S4 Volumetric water content vs. TDR data for a vertical TDR probe used for TDR probe calibration. Fig. S5 δ2H of soil water by depth measured at Barro Colorado Island (data from Meinzer et al., 1999). Fig. S6 Parameter sensitivity of ELM‐FATES soil water content and evapo‐transpiration. Fig. S7 Parameter sensitivity of ELM‐FATES stream discharge. Fig. S8 ELM‐FATES predicted soil water potential dynamics by depth over 1990–2018. Fig. S9 Leaf hydraulic vulnerability curves fitted to observed K leaf vs Ψleaf data for 21 tree species from Barro Colorado Island. Fig. S10 Percent loss of leaf hydraulic conductivity curves for ERD species, including curves fitted to K leaf vs Ψleaf as well as those based on scaling relationships with LMA and WSG. Fig. S11 Effective rooting depths (ERD) vs δ2Hxylem for each of the ERD model structures tested. Fig. S12 Correlation matrix between ERD and hydraulic traits. Fig. S13 Mortality rates for deciduous species across census intervals from 1985–2015 vs ERD. Fig. S14 Modeled effective rooting depths of deciduous species vs time spent beyond critical hydraulic threshold. Methods S1 Alternative structures for effective rooting depth mod [file NPH-231-1798-s001.pdf]

New Phytologist Supporting Information

Hydraulically-vulnerable trees survive on deep-water access during droughts in a tropical forest

Chitra-Tarak, Rutuja, Chonggang Xu, Salomón Aguilar, Kristina J. Anderson-Teixeira, Jeff Chambers, Matteo Detto, Boris Faybishenko, Rosie A. Fisher, Ryan G. Knox, Charles D. Koven, Lara M. Kueppers, Nobert Kunert, Stefan J. Kupers, Nate G. McDowell, Brent D. Newman, Steven R. Paton, Rolando Pérez, Laurent Ruiz, Lawren Sack, Jeffrey M. Warren, Brett T. Wolfe, Cynthia Wright, S. Joseph Wright, Joseph Zailaa, Sean M. McMahon

Article acceptance date: 29 April 2021

The following Supporting Information is available for this article:

Figure S1 Observed relationship between GPP and VPD at Barro Colorado Island.
Figure S2 Seasonal variation in estimated standardised LAI for 29 species at Barro Colorado Island.
Figure S3 Ensembles of soil hydraulic conductivity variation by depth for Conrad Trail Stream Catchment, Barro Colorado Island. Data from Godsey *et al.* (2004).
Figure S4 Volumetric water content vs. TDR data for a vertical TDR probe used for TDR probe calibration.
Figure S5 δ^2H of soil water by depth measured at Barro Colorado Island. Data from Meinzer *et al.* (1999).
Figure S6 Parameter sensitivity of ELM-FATES soil water content and evapo-transpiration.
Figure S7 Parameter sensitivity of ELM-FATES stream discharge.
Figure S8 ELM-FATES predicted soil water potential dynamics by depth over 1990-2018.
Figure S9 Leaf hydraulic vulnerability curves fitted to observed K_{leaf} vs. Ψ_{leaf} data for 21 tree species from Barro Colorado Island.
Figure S10 Percent loss of leaf hydraulic conductivity curves for ERD species, including curves fitted to K_{leaf} vs. Ψ_{leaf} as well as those based on scaling relationships with leaf mass area and wood specific gravity.
Figure S11 Effective rooting depth (ERD) versus δ^2H_{xylem} for each of the ERD model structures tested.
Figure S12 Correlation matrix between ERD and hydraulic traits.
Figure S13 Mortality rates for deciduous species across census intervals from 1985-2015 versus ERD.
Figure S14 Modeled effective rooting depths of deciduous species versus time spent beyond critical hydraulic threshold.

Table S1 ELM-FATES parameters used to generate ensembles, with description, prescribed global ranges, rationale for the choice of ranges and references, as well as ranges for best-fit ensembles.
Table S2 QA/QC procedure applied to eddy covariance fluxes.
Table S3 Above-ground hydraulic traits data from Wolfe *et al.* (2019) and Wolfe *et al.* (2021) used for comparison with ERD.
Table S4 Leaf vulnerability curve parameters A & B, $K_{\mathrm{max,leaf}}$ and $\Psi_{\mathrm{20,leaf}}$ by source (data or model) for the 29 species with ERD estimates.

Methods S1 Alternative structures for effective rooting depth model.
Methods S2 Statistics for identifying best-fit ERD.
Methods S3 Processing of forest data for growth estimates.
Methods S4 Leaf Area Index calculations.
Methods S5 Details of the ELM-FATES model.
Methods S6 Details for ELM-FATES model parameterization.
Methods S7 ELM-FATES calibration.

Dataset S1 Microclimatic and flux tower data.
Dataset S2 Leaf hydraulic conductivity and vulnerability to cavitation.
Dataset S3 Wood specific gravity (WSG) and leaf mass area (LMA).
Dataset S4 Leaf deciduousness categories.
Dataset S5 Stream discharge from the Conrad catchment.
Dataset S6 Volumetric water content.
Dataset S7 Stem maximum hydraulic conductivity and vulnerability to cavitation.
Dataset S8 Leaf turgor loss point.
Dataset S9 Above-ground hydraulic safety margins.

Notes S1 ERD model structure selection.
Notes S2 Additional results for exposure to water-stress.

Figure S1 Scatterplot of mean midday (11:00 am to 15:00 pm) gross primary productivity (GPP) and mean midday vapor pressure deficit (VPD) measured at the eddy flux tower in Barro Colorado Island over 2012-2017. Red line shows the fit from a polynomial model described in inset.

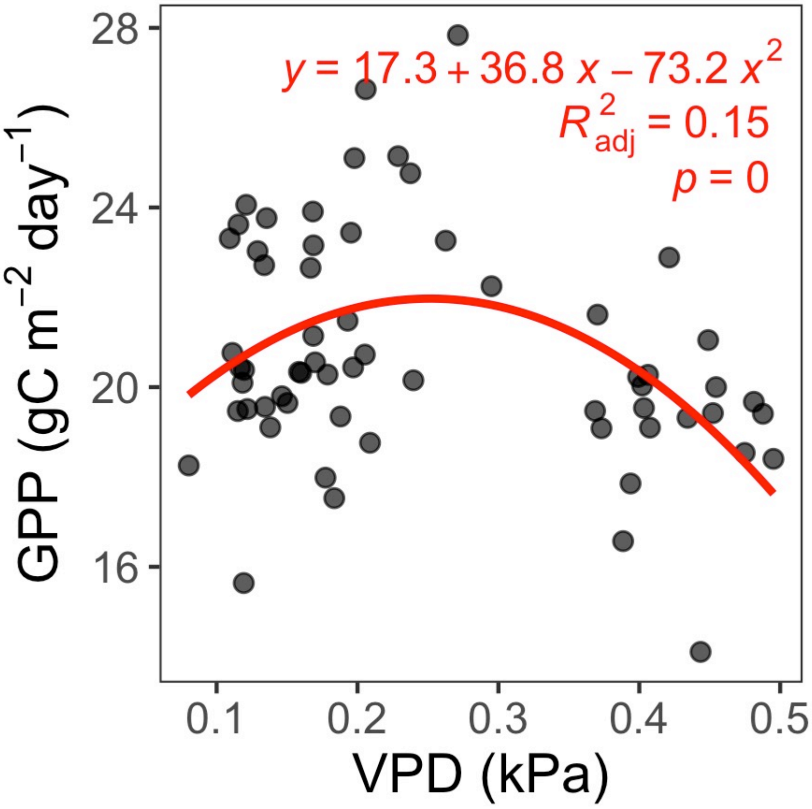

Figure S2 Seasonal variation in standardised LAI (range 0-1) for 29 species at Barro Colorado Island (panels). Species are color-coded by leaf habit as defined by expert botanists.

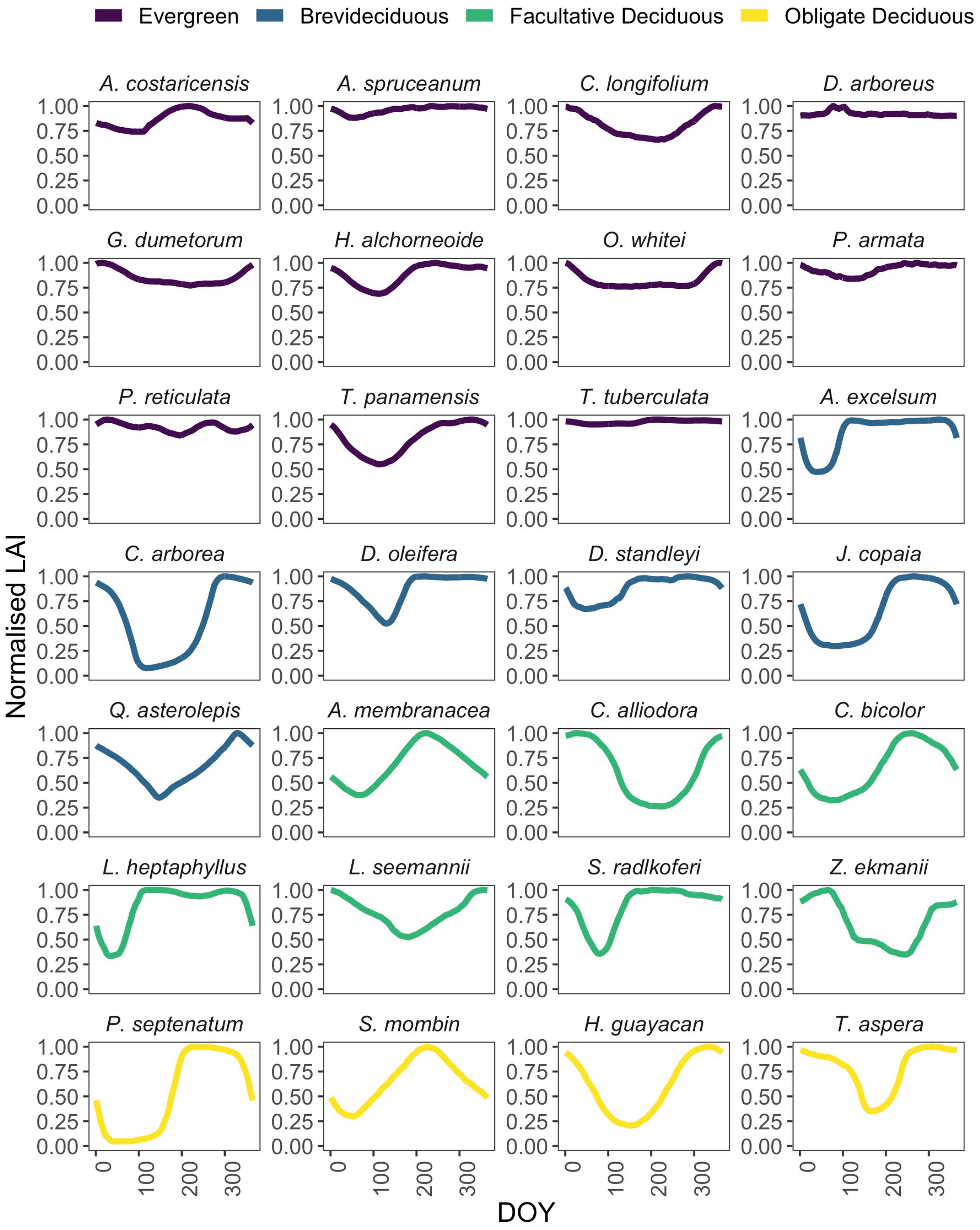

Figure S3 Generated 5000 curves of soil hydraulic conductivity variation by depth based on Latin Hypercube Sampling within 95% CI of observed data at depths of 12.5 cm and 60 cm for Conrad Trail Stream Catchment (Godsey *et al.*, 2004), assuming a linear decline between those depths, and values before 12.5 and after 60 cm as that of 12.5 cm and 60 cm, respectively. Only 100 curves are shown for clarity. Note the log scale on horizontal axis.

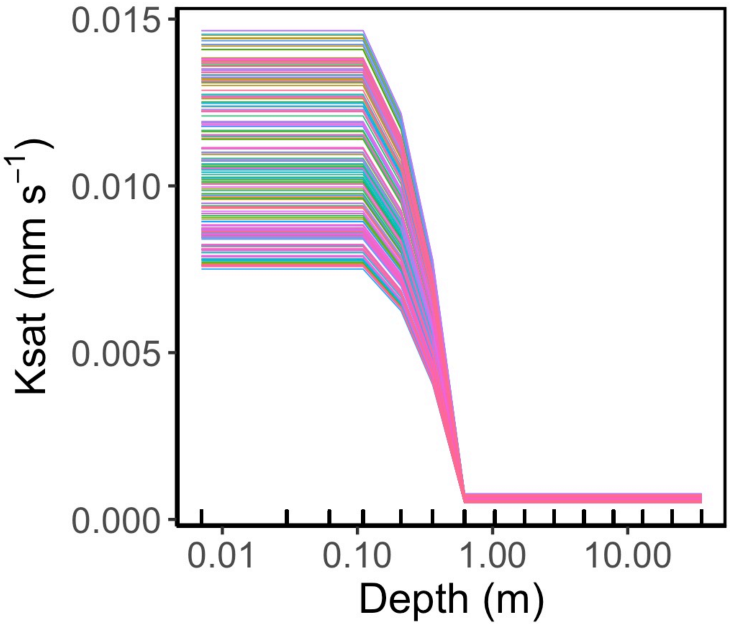

Figure S4 Volumetric water content vs. TDR data for a vertical TDR probe (0-15 cm depth) at the base of the eddy flux tower near the 50-ha plot at BCI.

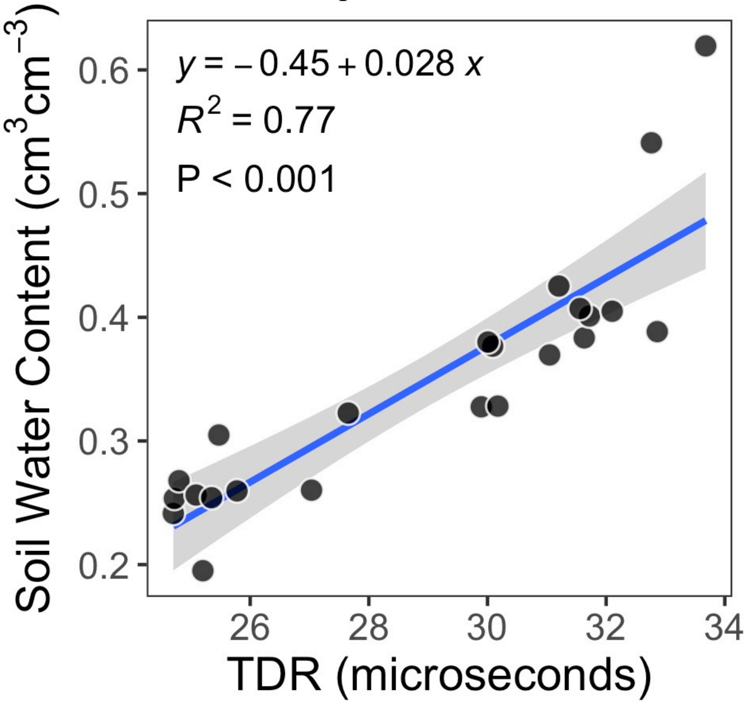

Figure S5 Stable isotopic ratio ($\delta^2 H$) of soil water by depth measured at Barro Colorado Island in March 1997. Groundwater sampled at the sources of springs on BCI during this period yielded an average $\delta^2 H$ value of –60 ‰ (not shown). Data from Meinzer *et al.* (1999). $\delta^2 H$ to depth relationship was particularly uncertain for $\delta^2 H$ values > -40 ‰. Two species, *Guapira standleyana* (-28.9 $\pm$ 3.7; mean $\pm$ SE) and *Spondias radlkoferi* (-33.5 $\pm$ 2.6), in Meinzer *et al.* (1999) dataset had $\delta^2 H_{xylem}$ values > -40 ‰. For example, *Guapira standleyana*, with $\delta^2 H_{xylem}$ value of -28.9 $\pm$ 3.7 could have a source-water depth anywhere between 10 to 50 cm. *Spondias radlkoferi* is also typically leafless in March-April. These two species were not included in the ERD model-data comparison.

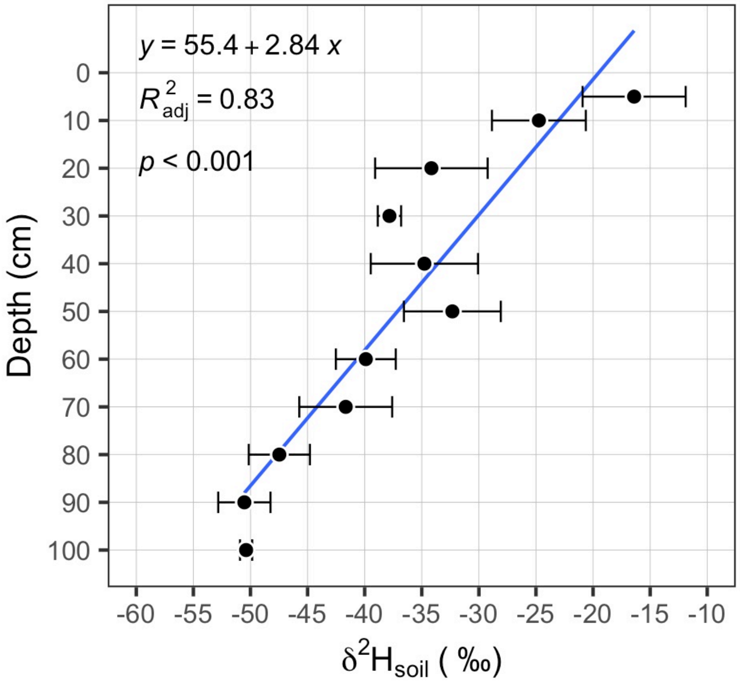

Figure S6 Parameter sensitivity of ELM-FATES soil water content (that is, VWC, **a**-**b**) and evapotranspiration (ET, **c**-**d**) is shown for two representative sample months, as sensitivity for a particular parameter varies depending on the chosen month. Horizontal axes show global ranges for a given parameter. Parameters are color coded and are consistent across panels. Each point represents a simulation among the 5000 ensemble-member runs. See Table S1 for description, global ranges and chosen ranges for parameters.

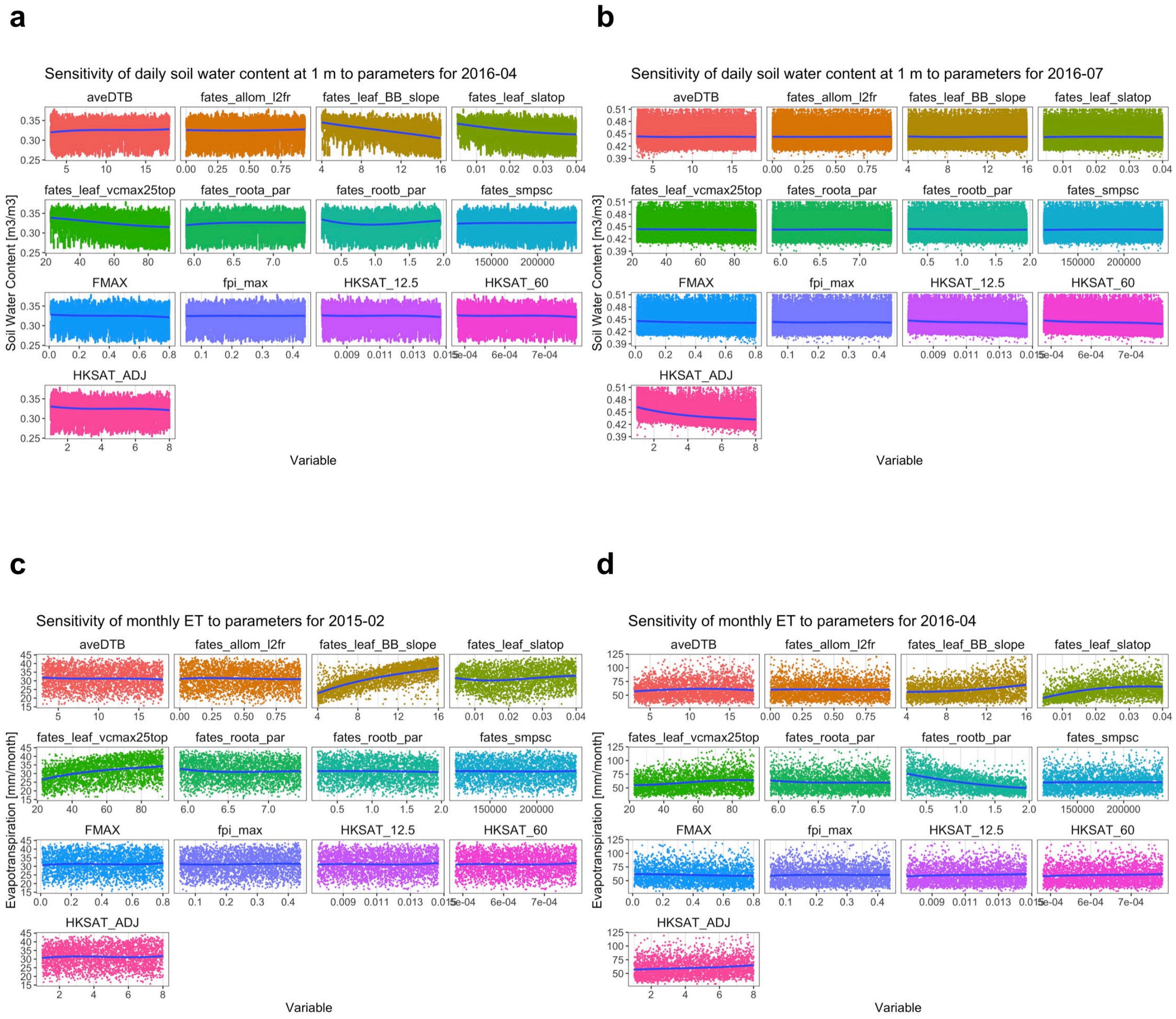

Figure S7 Parameter sensitivity of ELM-FATES stream discharge (QRUNOFF) is shown for four sample months (**a**-**d**), as sensitivity for a particular parameter varies depending on the chosen month. Horizontal axes show global ranges for a given parameter. Parameters are color coded and are consistent across panels. Each point represents a simulation among the 5000 ensemble-member runs. See Table S1 for description, global ranges and chosen ranges for parameters.

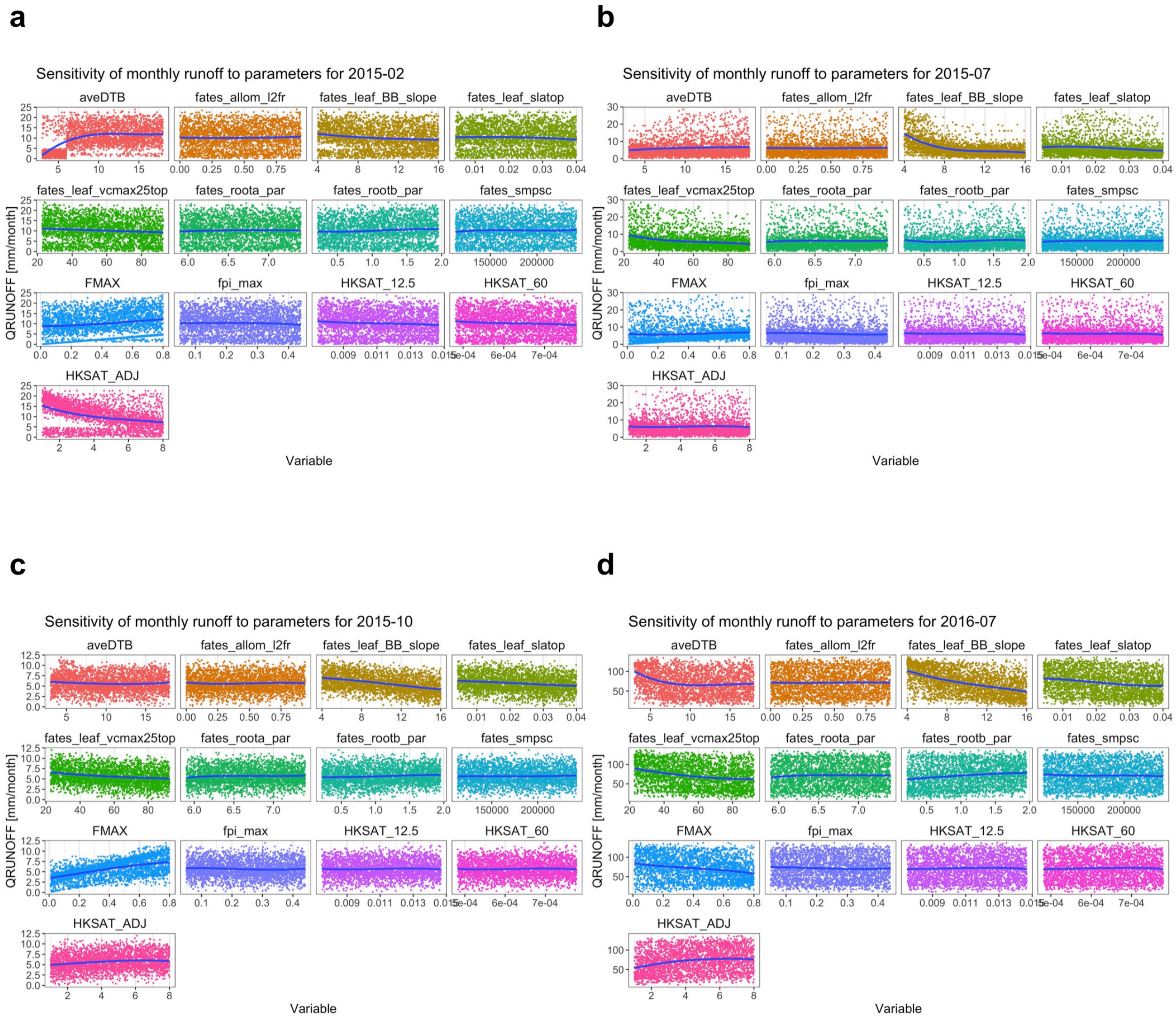

Figure S8 Daily mean soil water potential ($\Psi_{\mathrm{soil},z}$) by depth z (in m; panels) over 1990-2018 predicted from each of the 100 best-fit ensembles (colored lines). Vertical gray lines demarcate median dates of the tree demographic censuses at the BCI 50 ha plot.

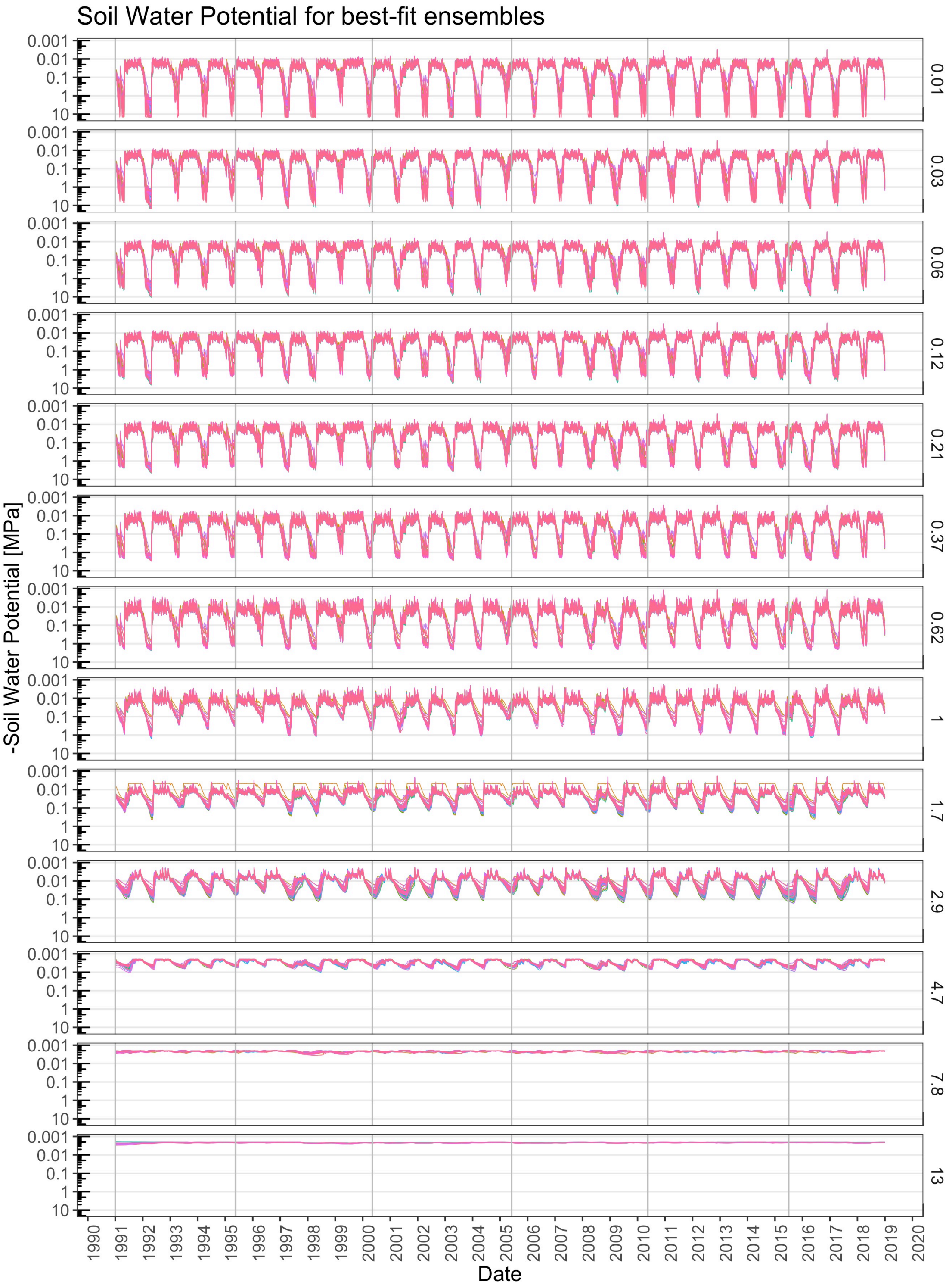

Figure S9 Leaf hydraulic vulnerability curves fitted to observed K_{leaf} vs. Ψ_{leaf} data for 21 tree species from BCI–in absolute terms (**a**, **b**), as well as, in terms of percent loss of conductivity (**c**, **d**). Each line represents a species, color coded by wood specific gravity (WSG) (**a**, **c**), or leaf mass area (LMA) (**b**, **d**), with blue tones indicating smaller values of WSG or LMA and red tones indicating larger values.

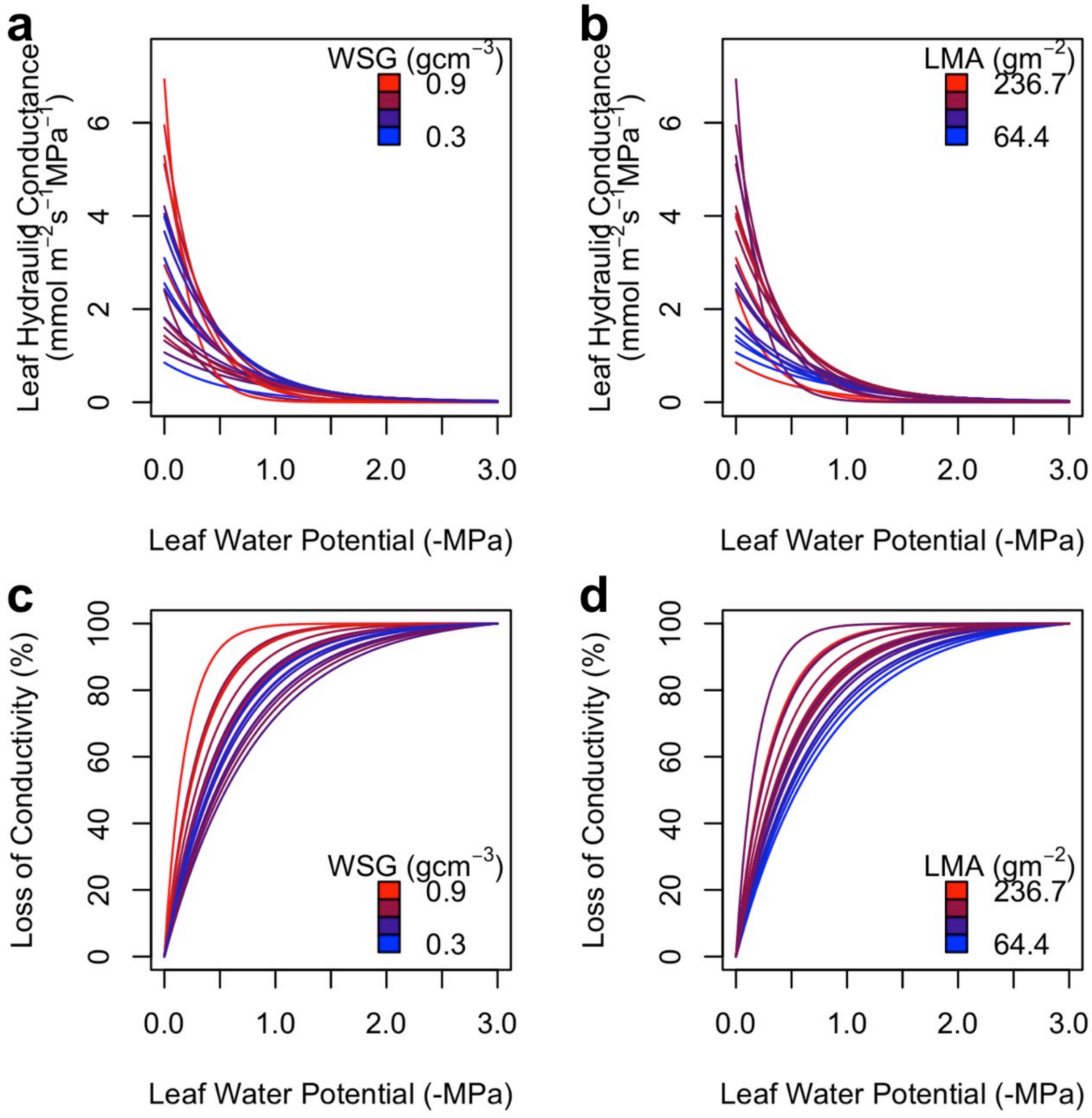

Figure S10 Percent loss of leaf hydraulic conductivity curves for species with ERD estimates. Each line represents a species, color coded by source of the curve, with those fitted to observed data for K_{leaf} vs. Ψ_{leaf} in yellow (data) and those based on the scaling relationships with leaf mass area and wood specific gravity in gray (model).

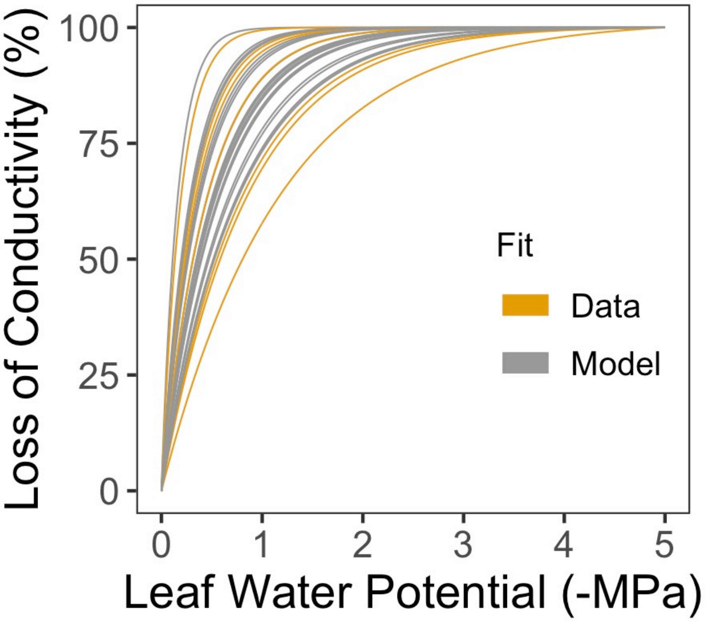

Figure S11 Effective rooting depth versus $\delta^2 H_{\text{xylem}}$ for each of the ERD model structures tested (panels). For each ERD model structure only those species have an estimate of ERD that have passed the test of Pearson's $r > 0.71$ during ERD parameterisation. The number of species with an ERD estimate as well as $\delta^2 H$ for each model structure are shown in inset, along with the goodness-of-fit (R^2) and p-values for a linear relationship between ERD and $\delta^2 H_{\text{xylem}}$. ERD models in panels **a-e** correspond to Eq. (S1)-(S6), respectively.

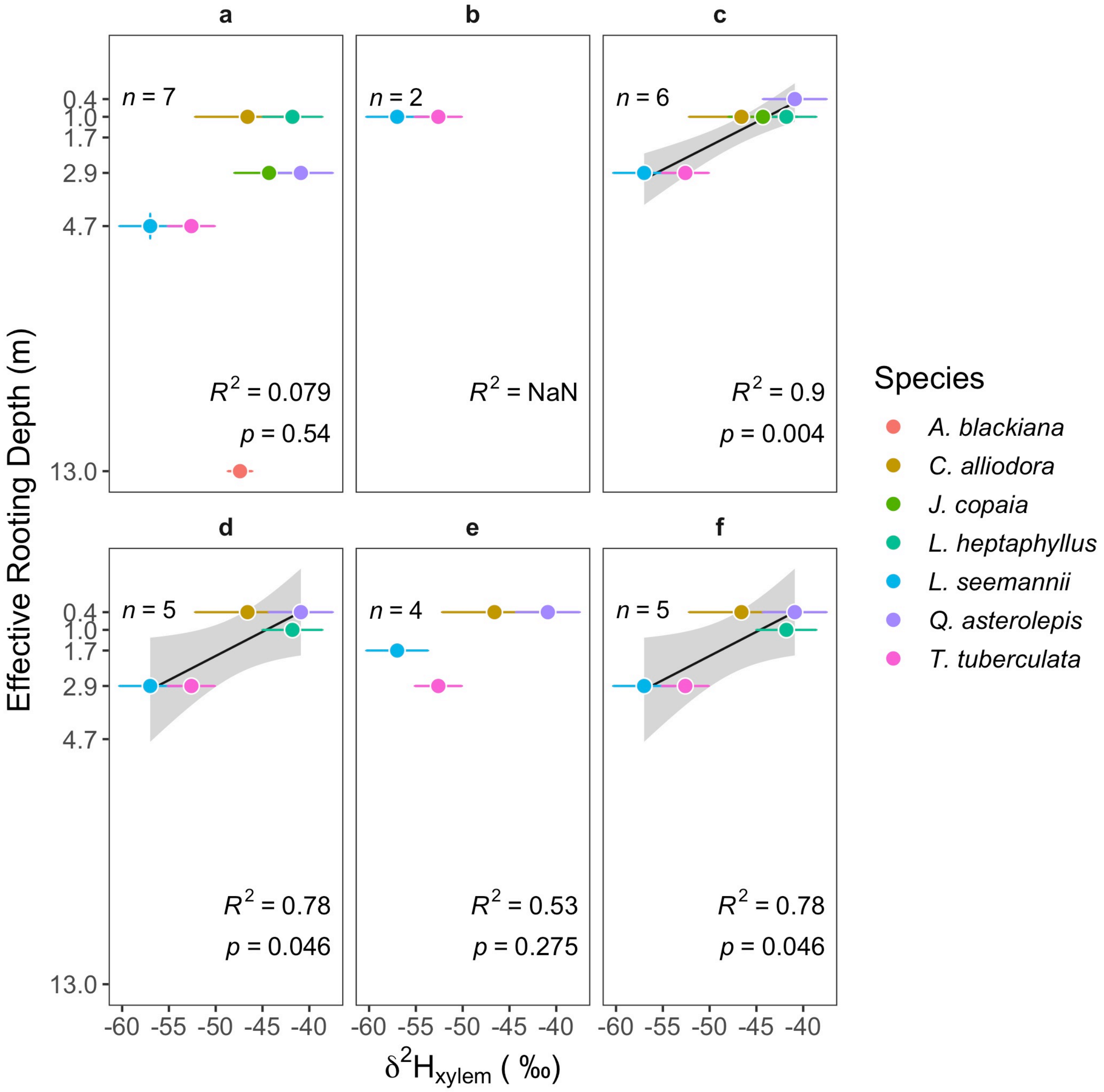

Figure S12 Pairwise Spearman's correlations (upper triangle) and corresponding linear regressions (lower triangle) among modelled Effective rooting depth (ERD) and hydraulic properties for seven canopy species found on Barro Colorado Island (Panama). Spearman's correlation coefficients and significance levels are shown (upper triangle; '∗∗' $p < 0.01$, '∗' $p < 0.05$, '.' $p < 0.1$).

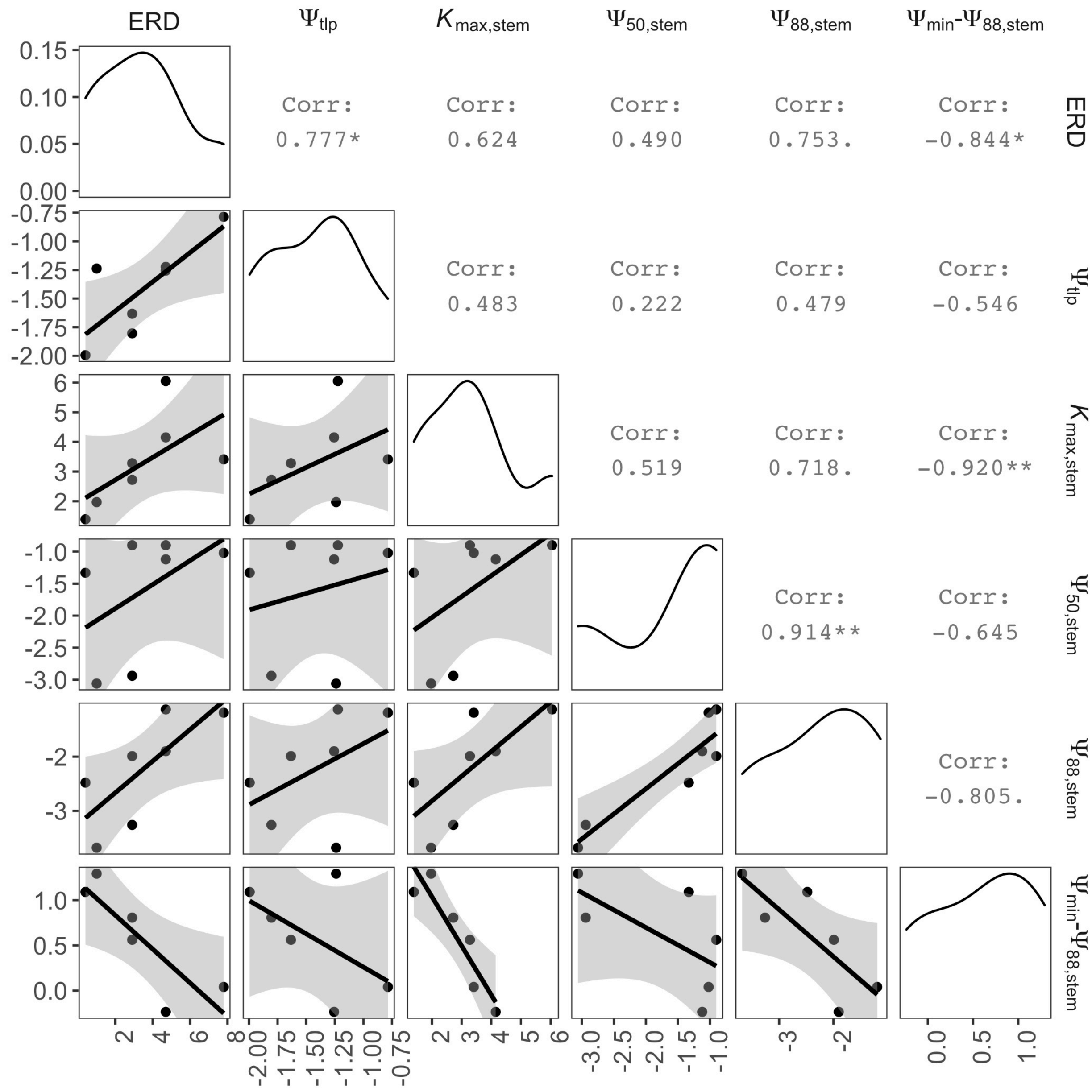

Figure S13 Mortality rates across census intervals from 1985-2015 for species (circles) of various deciduous leaf-habit categories (colors) plotted against ERD. R^2 and significance levels for linear model fits are given in panel insets. Census-intervals with a superscript $*$ and $**$ are shown for comparison with a corresponding figure for evergreen species (Fig. 8 in the main text) and indicate intervals in which significant mortality was explained by ERD among evergreen species at α = 0.1 and α = 0.05, respectively.

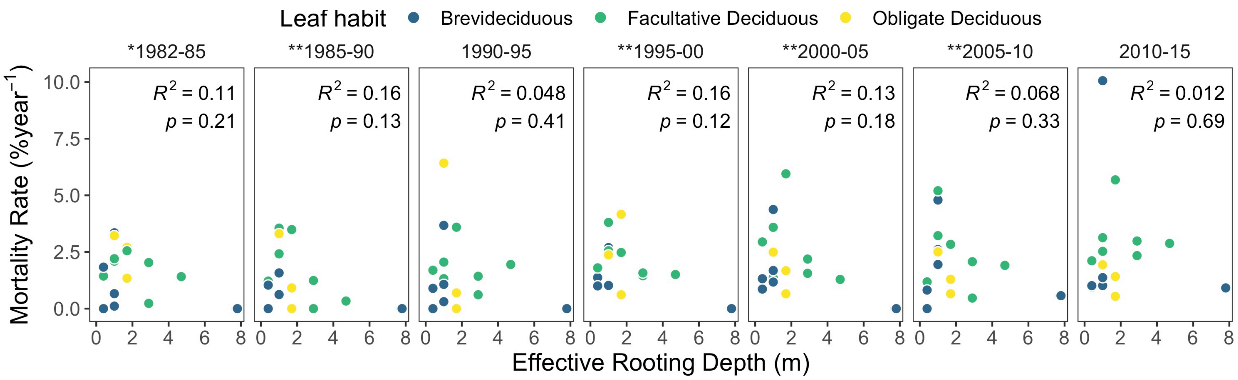

Figure S14 Modeled effective rooting depth (ERD; horizontal-axis) versus time spent beyond critical hydraulic threshold (vertical axis) by census interval (colored bars) for deciduous species in the mortality analyses (Fig. S13). Each bar represents the average time species of the same ERD spent beyond species-specific critical hydraulic thresholds in a given interval, that is, the proportion of days for which $\Psi_{\mathrm{soil},z=\mathrm{ERD}}$ was more negative than species Ψ_{crit}, defined as $\Psi_{20,\mathrm{leaf}}$, and where z is the soil depth matching species ERD. Standard error of the mean are shown over each bar. Note the squared y-axis scale.

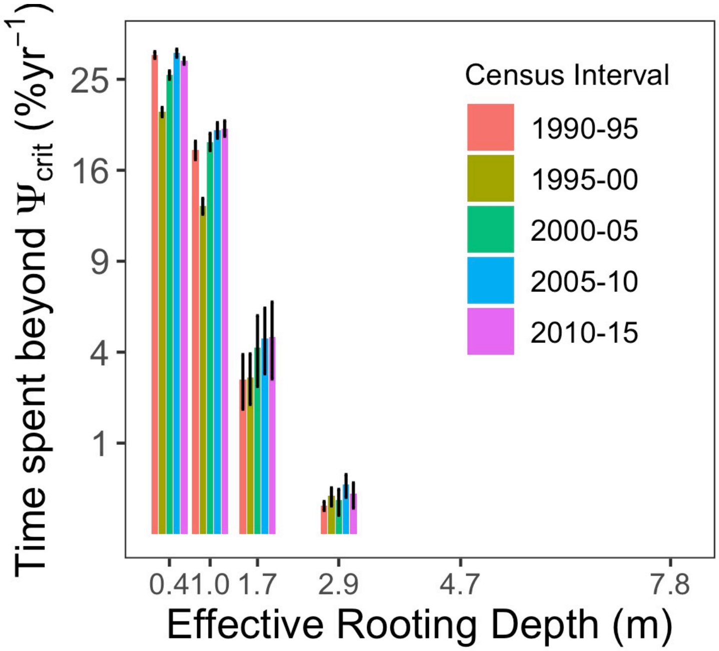

Table S1 ELM-FATES parameters used to generate ensembles, with parameter description, prescribed global ranges, rationale for the choice of ranges and references. Ranges for best-fit chosen ensembles are also given. Note that the first six parameters are specific to FATES.

Parameter	Description	Global mini-mum	Global maxi-mum	Best -fit mini-mum	Best-fit maxi mum	Units	Rationale and references
fates_leaf_BB_slope	stomatal slope parameter, as per Ball-Berry	4	16	7.8	15.9	unitless	The range of Ball Berry parameter fitted across several studies as compiled in Table 2 of (Medlyn *et al.*, 2012)
fates_leaf_slatop	Specific Leaf Area (SLA) at the top of canopy, projected area basis	0.0042	0.0400	0.0049	0.0399	m^2 gC^{-1}	Based on the observed range of Leaf Mass Area (LMA)–24.97 to 235.8 g m^{-2}– for individual tree variation across 51 tree species in Barro Colorado Island (this study), and assumption of 50% carbon content in biomass.
fates_leaf_vcmax25top	Maximum carboxylation rate of Rubisco at 25°C, canopy top	22.7	92.5	23.2	91.8	umol CO^2 m^2 s^{-1}	Observed range of V_{cmax} at 25°C for tropical tree species under relative radiation of 50% or greater, thereby excluding highly shaded leaves. Values derived with a conversion specific for CLM model are used. (Ali *et al.*, 2015)
fates_roota_par	ELM rooting distribution parameter	5.94	7.4	5.9	7.38	m^{-1}	Parameter a in Eq. 2 in (Zeng, 2001) that regulates the shape of the rooting profile. Range corresponds to this parameter specified for BATS (or IGBP) land cover classified Deciduous Broadleaf Trees (5.9 m^{-1} and Evergreen Broadleaf Trees (7.4 m^{-1}) as given in Table 1 of (Zeng, 2001).
fates_rootb_par	ELM rooting distribution	0.217	1.956	0.756	1.951	m^{-1}	Parameter b in Eq. 2 in (Zeng, 2001) that regulates the depth of the rooting profile. Chosen

	parameter						range of *b* is derived using this equation so as to fit the observed range of rooting depth (d_r) of 2 - 18 m for Tropical Deciduous Forest (mean ± Standard Error (SE); 3.7 ± 0.5, *n* = 5 trees; min = 2, max = 4.7) and Tropical Evergreen Forest (mean ± S.E.; 7.3 ± 0.5, n = 3 trees and 3 communities; min = 2, max = 18 m) combined (Canadell *et al.*, 1996). Besides the direct observation of roots at 18 m included by (Nepstad *et al.*, 1994) in Paragominas, eastern Amazonia that is included in the above study; in Tapajos, eastern Amazonia water extraction by roots was also inferred up to 18 m. (Davidson *et al.*, 2011)
fates_smpsc	Soil water potential at full stomatal closure	113000	242000	113294	236058	mm	Based on observed range of -1.13 to -2.42 MPa for leaf turgor loss point for individual tree variation across 49 BCI tree species. This study.
aveDTB	Distance to bedrock	3	18	4	17.4	m	Ben Turner, personal communication.
fmax	The maximum fractional saturated area	0.01	0.80	0.04	0.79	unitless	Empirical
HKSAT_ ADJ	Adjusting factor for soil hydraulic conductivity	1	8	3.1	8	unitless	To account for high macroporosity and direct flow paths in tropical soils that is not accounted for by small soil core samples (Tomasella & Hodnett, 1998; Kinner & Stallard, 2004; Broedel *et al.*, 2017)
HKSAT	Soil hydraulic conductivity profile	0.007 for =< 12.5 cm; 5.56e-05	0.014 for =< 12.5 cm; 8e-	0.008 for =< 12.5 cm; 5e-	0.015 for =< 12.5	mm s^{-1}	For Conrad catchment observed median Ksat (95% CI) for 12.5 cm depth is 38.3 mm hr^{-1} (25.4 - 51.2, n = 75), while for 60 cm depth 0.7 mm hr^{-1} (0.2 - 1.2, n = 40) (Godsey *et al.*, 2004).

		for >= 60 cm	04 for >= 60 cm	04 for >= 60 cm	cm; 8e-04 for >= 60 cm		For reference, a storm with 12.5 mm hr^{-1} rainfall intensity has a 0.2 probability of occurring in any given rainfall event.
fpi_max	Maximum interception fraction of precipitation	0.05	0.44	0.06	0.43	unitless	Based on throughfall data from (Zimmermann *et al.*, 2010) and precipitation data for BCI from STRI Physical Monitoring program, defined for precipitation events greater than 10 mm.

Table S2 QA/QC procedure applied to eddy covariance fluxes. w: vertical wind speed; t: air temperature; q: water vapor density; c: CO_2 density; u: horizontal wind speed. The bar indicates 30-min averaging and the carat indicates fluctuation ($\hat{x} = x - \overline{x}$)

Variable	Unit	Criterion
Rain	mm	During and 30 minutes after
Temperature flux	C m^{-2} s^{-1}	> -0.13 & < 0.51
H_2O flux	mmol m^{-2} s^{-1}	> -5 & < 30
CO_2 flux	mumol m^{-2} s^{-1}	> -60 & < 20
Turbulent intensity	-	< 5
Fiction velocity	m s^{-1}	> 0.15
H_2O 4th moment	mmol m^{-2}	< 55
CO_2 4th moment	mumol m^{-2}	< 55

Table S3 Minimum leaf water potential Ψ_{min}, leaf turgor loss point Ψ_{tlp}, stem maximum hydraulic conductivity $K_{max,stem}$ and stem vulnerability to cavitation $\Psi_{88,stem}$ for seven species with ERD estimates. Ψ_{min} is sourced from Wolfe *et al.* (2019), while Ψ_{tlp}, $K_{max,stem}$, $\Psi_{50,stem}$ and $\Psi_{88,stem}$ are sourced from Wolfe *et al.* (2021). Ψ_{min}, Ψ_{tlp}, $\Psi_{50,stem}$ and $\Psi_{88,stem}$ are in MPa, while $K_{max,stem}$ in kg m^{-1} s^{-1} MPa^{-1}.

Genus	Species	Family	Ψ_{min}	TLP	$K_{max,stem}$	$\Psi_{50,stem}$	$\Psi_{88,stem}$
Anacardium	excelsum	Anacardiacea	-1.39	-1.99	1.39	-1.33	-2.48
Apeiba	membranacea	Tiliaceae	-1.43	-1.63	3.28	-0.90	-1.99
Cordia	alliodora	Boraginaceae	-2.39	-1.24	1.97	-3.06	-3.68
Guatteria	dumetorum	Annonaceae	-2.14	-1.26	4.15	-1.12	-1.90
Luehea	seemannii	Tiliaceae	-2.45	-1.80	2.72	-2.94	-3.26
Pseudobombax	septenatum	Bombacaceae	-1.15	-0.79	3.41	-1.02	-1.19
Spondias	radlkoferi	Anacardiacea	NA	-1.22	6.05	-0.90	-1.13

Table S4 Leaf vulnerability curve parameters *A* & *B*, $K_{max,leaf}$ and $\Psi_{20,leaf}$ for the 29 species with ERD estimates. Species for which curves were fitted to observed data for K_{leaf} vs. Ψ_{leaf} are identified with column Source as 'Data', while species for which curves were obtained based on trait-proxies are identified as 'Model'.

Genus	Species	Family	*A*	*B*	$K_{max,leaf}$	$\Psi_{20,leaf}$	Source
Anacardium	excelsum	Anacardiacea	1.4822	0.8415	1.48	-0.27	Data
Calophyllum	longifolium	Clusiaceae	2.2918	3.1691	2.29	-0.07	Data
Dipteryx	oleifera	Fabaceae:Pap	5.9680	5.1644	5.97	-0.04	Data
Luehea	seemannii	Tiliaceae	6.0308	2.9551	6.03	-0.08	Data
Poulsenia	armata	Moraceae	3.1311	2.2162	3.13	-0.10	Data
Quararibea	asterolepis	Bombacaceae	1.4618	1.1832	1.46	-0.19	Data
Spondias	radlkoferi	Anacardiacea	1.6387	1.2543	1.64	-0.18	Data
Alchornea	costaricensis	Euphorbiacea	1.0035	1.3383	1.00	-0.17	Model
Apeiba	membranacea	Tiliaceae	3.2784	1.8654	3.28	-0.12	Model
Aspidosperma	spruceanum	Apocynaceae	26.6792	6.2107	26.68	-0.04	Model
Casearia	arborea	Flacourtiace	1.2350	1.3430	1.23	-0.17	Model
Cordia	alliodora	Boraginaceae	2.0372	1.5153	2.04	-0.15	Model
Cordia	bicolor	Boraginaceae	3.9529	1.9081	3.95	-0.12	Model
Dendropanax	arboreus	Araliaceae	3.1282	1.7609	3.13	-0.13	Model
Drypetes	standleyi	Euphorbiacea	5.1217	2.5969	5.12	-0.09	Model
Guapira	standleyana	Nyctaginacea	1.7657	1.4649	1.77	-0.15	Model
Guatteria	dumetorum	Annonaceae	3.1220	1.7725	3.12	-0.13	Model
Hieronyma	alchorneoide	Euphorbiacea	3.8604	2.2041	3.86	-0.10	Model
Jacaranda	copaia	Bignoniaceae	2.8856	1.7336	2.89	-0.13	Model
Lonchocarpus	heptaphyllus	Fabaceae:Pap	4.9740	2.8009	4.97	-0.08	Model
Ocotea	whitei	Lauraceae	4.2595	1.9960	4.26	-0.11	Model
Pouteria	reticulata	Sapotaceae	4.5825	3.3883	4.58	-0.07	Model
Pseudobombax	septenatum	Bombacaceae	3.9295	1.9559	3.93	-0.11	Model
Spondias	mombin	Anacardiacea	3.6633	1.8712	3.66	-0.12	Model
Handroanthus	guayacan	Bignoniaceae	1.0418	2.6882	1.04	-0.08	Model
Tetragastris	panamensis	Burseraceae	12.1515	3.5370	12.15	-0.06	Model
Trattinnickia	aspera	Burseraceae	4.1726	1.9868	4.17	-0.11	Model
Trichilia	tuberculata	Meliaceae	6.8407	3.3081	6.84	-0.07	Model
Zanthoxylum	ekmanii	Rutaceae	1.2795	1.3145	1.28	-0.17	Model

Methods S1 Alternative structures for effective rooting depth model.

Below we present all the alternative models for effective rooting depth (ERD). Daily average growth, $\hat{G}_{s,t|z}$ for species s, in the census interval t is described as follows, in which K_{leaf}, and thus FLC_{leaf}, is driven by soil water dynamics at depth z for a given hydrological realisation h:

$$\hat{G}_{s,t|z} = \beta_{0,s|z,h} + \beta_{1,s|z,h}(\frac{1}{n_t}\sum_{i=1}^{n_t} \text{FLC}^*_{\text{leaf},s,i|z,h}) + \epsilon_{s,t|z,h} \quad (S1)$$

$$\hat{G}_{s,t|z} = \beta_{0,s|z,h} + \beta_{1,s|z,h}(\frac{1}{n_t}\sum_{i=1}^{n_t} \text{FLC}^*_{\text{leaf},s,i|z,h} + \widehat{\text{VPD}}^*_i) + \epsilon_{s,t|z,h} \quad (S2)$$

$$\hat{G}_{s,t|z} = \beta_{0,s|z,h} + \beta_{1,s|z,h}(\frac{1}{n_t}\sum_{i=1}^{n_t} \text{FLC}^*_{\text{leaf},s,i|z,h}\,\widehat{\text{VPD}}^*_i) + \epsilon_{s,t|z,h} \quad (S3)$$

$$\hat{G}_{s,t|z} = \beta_{0,s|z,h} + \beta_{1,s|z,h}(\frac{1}{n_t}\sum_{i=1}^{n_t} \text{FLC}^*_{\text{leaf},s,i|z,h}\,\text{LAI}^*_{s,doy|i}) + \epsilon_{s,t|z,h} \quad (S4)$$

$$\hat{G}_{s,t|z} = \beta_{0,s|z,h} + \beta_{1,s|z,h}(\frac{1}{n_t}\sum_{i=1}^{n_t} \text{FLC}^*_{\text{leaf},s,i|z,h} + \widehat{\text{VPD}}^*_i)\text{LAI}^*_{s,doy|i}) + \epsilon_{s,t|z,h} \quad (S5)$$

$$\hat{G}_{s,t|z} = \beta_{0,s|z,h} + \beta_{1,s|z,h}(\frac{1}{n_t}\sum_{i=1}^{n_t} \text{FLC}^*_{\text{leaf},s,i|z,h}\,\widehat{\text{VPD}}^*_i\text{LAI}^*_{s,doy|i}) + \epsilon_{s,t|z,h} \quad (S6)$$

where n_t are the total number of days in census interval t, the superscript * indicates that the variable has been standardized between 0 and 1, and ϵ is the model error term. $\widehat{\text{VPD}}$ is the predicted GPP from a locally derived polynomial relationship between gross primary productivity (GPP) and VPD, so as to account for the non-linear impact of VPD on growth. See the ERD model description in the main text for other details.

Methods S2 Statistics for identifying best-fit ERD.

For each ERD model structure (Eqs. (S1)-(S6)), species-specific best-fit ERD was identified as follows: for example, for the chosen model (Eq. (S3)), for a species s and hydrological realization h for soil water potential $\Psi_{\text{soil},z}$ dynamics, we obtained Akaike's Information Criterion (AIC) and Goodness-of-fit (R^2) for each modeled soil layer depth z. We used AIC as the first selection criterion to ensure that the fitted growth values from Eq. (S3) are within the confidence interval of the error distribution of observed growth (as the same model Eq. (S3) was being compared for each h, number of model parameters were the same). For each h, we retained those depths with the maximum likelihood of growth (similar AIC based support, see below) filtering out the rest. However, AIC does not ensure that fitted values will have a positive trend with growth. We therefore used R^2 between observed and fitted growth values as a goodness-of-fit measure and retained depths associated with a relatively high threshold R^2 value of 0.5; after filtering out depths associated with negative correlations r, if any. Finally, among these we chose the depth with the highest R^2 per h. Results were not sensitive to inclusion of this

final step of retaining only a single depth as the best-fit (not shown). For a species s, we defined effective rooting depth (ERD) as the median of the best-fit depth over all h, which can be described as:

$$\text{ERD}_s = \underset{\forall\, h \in H}{\text{med}}(z \mid (R^2_{s|z,h} = \underset{\forall\, z \in Z}{\max}(R^2_{s|z,h} | \Delta_{s|z,h} \leq 2)) \cap R^2_{s|z,h} \geq 0.5 \cap r_{s|z,h} \geq 0) \qquad (S7)$$

where

$$\Delta_{s|z,h} = \text{AIC}_{s|z,h} - \underset{\forall\, z \in Z}{\min}(\text{AIC}_{s|z,h}) \qquad (S8)$$

and

$$\text{AIC}_{s|z,h} = -2\log(\mathcal{L}(\hat{\beta}_{s|z,h} | G_s)) - 2\text{k} \qquad (S9)$$

where r is the correlation, and R^2 the Goodness-of-fit, between observed growth G and estimated growth $\hat{G}$. $\mathcal{L}$ is the likelihood function, $\hat{\beta}$ is the maximum-likelihood estimate, G the observed growth with k observations (or, census intervals t). H = (1, .., 100), the 100 best-fit hydrological realizations and Z = (0.4, 1, 1.7, 2.9, 4.7, 7.8, 13) m. We removed finely resolved soil layers <0.21 m and averaged $\Psi_{\text{soil},z}$ for depths 0.21, 0.37 and 0.62 (=0.4) m as sufficient resolution for model validation.

Methods S3 Processing of forest data for growth estimates.

We used tree diameter recensus data from the Barro Colorado 50-ha plot (Condit *et al.*, 2019). We selected trees for which the height of the diameter measurements were unchanged across all censuses (1990, 1995, 2000, 2005, 2010, 2015). For each individual tree (i), we calculated absolute growth rates $\grave{G}_{i,t}$ (cm yr^{-1}) for each of the five census intervals t as the difference between tree dbh measured in successive censuses prorated by the inter-census interval based on the exact date of measurements. We eliminated positive outliers as any growth rates amounting to >75 mm yr^{-1}, as this was the highest rate observed with confidence for the fastest growing species (Condit, 2017). Negative outliers were removed based on known measurement error, σ_e = 0.006214d + 0.903, where d is dbh. Any stem whose later dbh measurement fell <4σ_e the earlier measurement was removed (Condit, 2017). As tree growth rates vary with size (Muller-Landau *et al.*, 2006), to keep growth rates comparable across census intervals, we obtained residuals $\text{R}_{i,t}$ from a dbh model of tree growth based on a tree's dbh at the beginning of a census interval ($\text{R}_{i,t} = \grave{G}_{i,t} - f(d_{i,t})$). The diameter model of growth, $f(d_{i,t})$, a B-spline based polynomial equation with five degrees of freedom, was fitted to growth rates from all trees pooled together as there was insufficient species-specific data for large trees (R^2 = 0.27; p-value < 0.01). We retained only those trees that had $\text{R}_{i,t}$ estimates for all census intervals (total trees = 972) and standardized (centered and scaled) individual tree time series of $\text{R}_{i,t}$. For each species s we then obtained growth time series G_s as the time-series of medians of $\text{R}_{i,t}$ for the five census intervals t to use in the ERD model. G_s were obtained for only those species (n = 29) with complete records on at least three trees (median 10, max 111 trees per species).

Methods S4 Leaf Area Index calculations.

We generated an estimate of the mean seasonality curve for normalized leaf area index (LAI^*, range 0-1, unitless) for individual species as follows. We interpolated species-specific

weekly leaf-fall data collected at two similar sites in BCI (1985-2020) to a daily estimate of leaf mass fall per unit ground area (Wright & Cornejo, 1990). We estimated living canopy leaf mass per unit ground area present at time t, L[t], from mean leaf longevity in days, LL, and leaf mass fall per unit ground area at time t, F[t], as follows: L[t] = F[t+1] + F[t+2] + … + F[t + LL]. L[t] was averaged by DOY across years and divided by the max value to obtain the species-specific seasonality curve in LAI* (range 0-1, unitless). (Fig. S2).

For 13 species, an estimate of leaf lifespan was available from direct observations at a dry and wet site in Panama (Osnas *et al.*, 2018). For the remaining species leaf lifespan was gap-filled with the following preference order depending on data availability: genus-level mean (n = 1), family-level mean (n = 7), site-level mean (n = 6 deciduous species using data from the site Parque Natural Metropolitano situated on the drier side of the rainfall gradient along the Isthmus of Panama). For one species, leaf lifespan was predicted from leaf mass area (LMA) ($\overline{lifespan} = 3.18 + 2.72LMA$; $R^2 = 0.65$; $p = 0.0027$, $n = 11$).

Although deciduous leaf-habits are generally expected to be fully deciduous, at BCI there is a large within-species variation in the timing of leaf-drop and leaf-out and not all individuals of a deciduous species reach full deciduousness each year (Condit *et al.*, 2000). Our reconstruction of LAI* seasonality is based upon species leaf-fall data, which captures the within-species variation in the timing of leaf-drop. So, in our reconstruction the seasonal decline in LAI* appears to be more spread out and does not reach zero than that is one expected for an idealized individual representing each of the deciduous leaf habits. Greater uncertainty in leaf-lifespan for deciduous species may have compounded this issue further. We did not normalize the deciduous species' LAI* curves to reach zero as it may further distort the shape of the LAI* curve shifting a larger period under a lower LAI*.

Methods S5 Details of the ELM-FATES model.

In ELM, soil water is predicted from a multi-layer model, in which the vertical soil moisture transport is governed by infiltration, surface and sub-surface runoff, gradient diffusion, gravity, canopy transpiration through root extraction, and interactions with groundwater (see Oleson *et al.* (2013) for process details and equations). FATES simulates size-structured tree community complete with individual tree rooting profiles. In our use of reduced-complexity configuration of ELM-FATES with a single plant functional type, ELM removes total community canopy transpiration as water-uptake from the soil layers in proportion to the total root fraction in each layer in the 1-D column. Note that we use the soil water potential dynamics obtained from ELM-FATES as an ecosystem-level outcome to drive our species-level, empirical ERD model. We thus believe that there is no conflict/circularity between tree-level rooting profiles or water uptake processes in ELM-FATES and the ERD model.

We ran ELM with FATES vegetation, in which the ELM model simulates interception, throughfall, canopy drip, infiltration, evaporation, surface runoff, subsurface drainage, redistribution within the soil column, and groundwater discharge and recharge so as to simulate changes in canopy water ΔW_{can}, surface water ΔW_{sfc}, soil water $\Delta W_{\mathrm{liq},z_i}$ at depth z_i and water in an unconfined aquifer ΔW_a (omitting processes relevant to snow, wetlands or lakes). Conservation of these terms is expressed as follows:

$$\Delta W_{\text{can}} + \Delta W_{\text{sfc}} + \sum_{i=1}^{15} \Delta W_{\text{liq},z_i} + \Delta W_a = \left(q_{\text{rain}} - E_v - E_g - q_{\text{over}} - q_{\text{h2osfc}} - q_{\text{drai}}\right)\Delta t \qquad (S10)$$

where, q_{rain} is rainfall, E_v is transpiration of the forest, E_g is ground evaporation, q_{over} is surface runoff, q_{h2osfc} is runoff from surface water storage, q_{drai} is subsurface drainage (all in mm H_2O) and Δt is the time-step (here, 1 hr).

Justification for the use of static stand structure: in static stand structure model, the model is started from an observed forest inventory and recruitment, growth and mortality are deactivated. Holding the stand structure static rather than allowing it to emerge from physiologically-mediated competition between trees removes a set of internal model feedbacks and thus the only differences between model simulations are driven by direct physiological effects of perturbed model parameters. This allows us to focus on the hydraulic and hydrological questions explored in this paper.

Methods S6 Details for ELM-FATES model parameterization.

In the Conrad Trail Stream catchment, in which the 50 ha plot is situated, soil hydraulic conductivity decreases with depth from 12.5 and 60 cm (Godsey *et al.* (2004); Table 1). We generated 5000 LHS samples within the 95% CI for the two depths and assumed a linear decline between each paired sample, while for depths <12.5 cm and >60 cm, we assumed the same value as that for depths 12.5 and 60 cm, respectively (Fig. S3).

For greater accuracy, instead of the ELM default pedo-transfer functions, we estimated parameters of soil retention curves using existing data for gravimetric water content (GWC) vs. Ψ_{soil} for depths of 0.15, 0.4 and 1 m (Kupers *et al.*, 2019). We converted GWC to volumetric (VWC, θ) using a measured bulk density value of 0.8 g cm^{-3} (see below). The Campbell (1974) empirical equation was fitted for all depths pooled together, as exploratory analyses indicated that differences in water retention among depths were nominal,

$$\Psi_{\text{soil}} = \Psi_e \left(\frac{\theta}{\theta_s}\right)^{-b} \qquad (S11)$$

where Ψ_e is the air-entry matric potential, θ_s is the saturated volumetric water content, and b, an index for soil pore-size distribution, respectively. Ψ_e, θ_s and b that best-fit the data were 200 (mm H_2O), 0.51 (cm^3 cm^{-3}) and 10 (unitless), respectively.

Methods S7 ELM-FATES calibration.

We calibrated ELM-FATES over 2012-2018 against three key fluxes and states in Eq. (10), namely, (1) evapotranspiration ET from the flux tower by the 50 ha plot ($\simeq E_v + E_g$ in Eq. (10); 2012-2017; Dataset S2), (2) local stream discharge q_{run} ($\simeq q_{\text{over}} + q_{\text{drai}}$; 2012-2018; see Paton (2019) and Dataset S5) and (3) soil volumetric water content (VWC) θ_z ($\simeq W_{\text{liq},z}$) from two sources: (i) a long-term (2012-2018) record of VWC $\theta_{0\text{-}0.15\text{ m}}$ averaged across three vertical time domain reflectometry (TDR) probes over depth 0-15 cm from three locations near the flux tower, and (ii) plot-wide snap-shot measurements of VWC during the dry season of 2015 and 2016 at depths of 0.15, 0.4 and 1 m (1299 samples covering all soil types and habitats; see

Dataset S6 and Kupers *et al.* (2019) for details; hereafter, $\theta_{0\text{-}0.15\ \mathrm{m}}$, $\theta_{0.4\ \mathrm{m}}$ and $\theta_{1\ \mathrm{m}}$ refer to plot-wide averages).

For ELM-FATES calibration we calculated an objective function for each of the 5000-member ensemble by equally weighting standardized RMSE between observations and simulations across all fluxes and states mentioned above. We describe the objective function O as:

$$\overline{O} = \frac{1}{6}\sum \mathrm{RMSE}^*(\mathrm{ET}, E_v + E_g) + \mathrm{RMSE}^*(q_{\mathrm{run}}, q_{\mathrm{over}} + q_{\mathrm{drai}}) + \mathrm{RMSE}^*(\theta_{0\text{-}0.15\ \mathrm{m}}, W_{\mathrm{liq},\overline{z}})$$
$$+\mathrm{RMSE}^*(\theta_{0.15\ \mathrm{m}}, W_{\mathrm{liq},0.12\mathrm{m}}) + \mathrm{RMSE}^*(\theta_{0.4\ \mathrm{m}}, W_{\mathrm{liq},0.37\ \mathrm{m}}) + \mathrm{RMSE}^*(\theta_{1\ m}, W_{\mathrm{liq},1\ \mathrm{m}}) \qquad (S12)$$

based on monthly sums for both ET and q_{run} and daily averages for θs. $W_{\mathrm{liq},\overline{z}}$ is the average of daily $W_{\mathrm{liq},z}$, where $z \in Z$; Z = (0.01, 0.03, 0.06, 0.12) m. Superscript * indicates that RMSE is standardized between 0 and 1 across the 5000 simulations.
Model fit to soil moisture dynamics by depth was one of our key priority. Preliminary analyses showed that fitting the model to all states and fluxes simultaneously captured soil moisture dynamics for shallower depths but not at 1 m depth. To ensure model fit at 1 m depth, we thus imposed a two-step procedure, wherein we first chose the 300 parameter ensemble members that minimized $\mathrm{RMSE}^*(\theta_{1\ \mathrm{m}}, W_{\mathrm{liq},1\ \mathrm{m}})$ (< 0.2), and among those 300 ensemble members we chose 100 with the lowest values for the objective function $\overline{O}$.

Dataset S1 Microclimatic and flux tower data.

The tower used for these measurements is 41 m above ground, on a plateau on BCI at the north west corner of the 50ha plot (Pau *et al.*, 2018). The eddy covariance system includes a sonic anemometer (CSAT3, Campbell Scientific, Logan, UT) and an open-path infrared CO_2/H_2O gas analyzer (LI7500, LiCOR. Lincoln, NE). Hi-frequency (10Hz) measurements were acquired by a datalogger (CR1000, Campbell Scientific) and stored on a local PC. Data were processed with a custom program using a standard routine described in (Detto *et al.*, 2010). QA/QC criteria for removing erroneous values are listed in Table S2. GPP was derived from daytime values of NEE by adding the corresponding mean daily ecosystem respiration obtained as the intercept of the light response curve (Lasslop *et al.*, 2010). The light curve was fitted on a 15-days moving window using a rectangular hyperbolic function (runs with friction velocity less than 0.4 m s^{-1} were excluded). In order to compute daily time integrated budgets, gaps were filled using Artificial Neural Network (Papale & Valentini, 2003) with hydro-meteorological inputs as predictors (soil moisture, solar radiation, temperature, VPD and air pressure, all measured on the same tower). To train the network, the dataset was randomly divided in a training set (70%), a validation set (15%) and a test set (15%). A two-layer feed-forward network with 10 sigmoid hidden neurons and linear output neurons, was trained using the Levenberg-Marquardt algorithm until the mean square error (MSE) of the validation set stopped improving (Hagan & Menhaj, 1994). Performance, in terms of MSE, was evaluated using the test set at the end of the training. This procedure was repeated 100 times to produce 100 estimates of GPP. Training multiple times generates different results due to different initial conditions and random sampling of the three sets. Ensemble was obtained as weighted average from the 100 ANN predictions using the MSE of the test set as weights according to:

$$f_i = \frac{\sum_k f_{ik}^{ANN} / MSE_{ik}}{\sum_k 1 / MSE_{ik}}$$

The ANN was implemented using the Neural Network Toolbox in Matlab 2014a.

Data can be requested from https://ameriflux.lbl.gov.

Dataset S2 Leaf hydraulic conductivity and vulnerability to cavitation.

K_{leaf} was measured under steady state and high irradiance following the Evaporative Flux Method (FEM) (Sack & Scoffoni, 2012) using a flowmeter (J. Zailaa et al., unpublished). Briefly, the leaf was excised under filtered deionised water and attached to a water source (graduated cylinder) using clear plastic tubing. The system includes tubing of a known resistance, separated upstream and downstream by two sensors measuring the transpiration-driven water flow pressure in the EFM system (Sack *et al.*, 2011). K_{leaf} is calculated as the ratio of the transpiration rate (E_{leaf}; mmol s^{-1}) to the difference in flow pressure (MPa) upstream and downstream from the resistance tubing, and further standardized to 25°C and by leaf area. At stability (CV < 5%), the leaf is left to equilibrate in plastic bags with moist paper towels and measured for Ψ_{leaf} using a Scholander pressure chamber. To construct vulnerability curves for each species, four different functions were fitted to species-specific K_{leaf} vs. Ψ_{leaf} data using the *anneal* optimization function in the R package likelihood: exponential without an intercept ($K_{leaf} = Ae^{-B\Psi_{leaf}}$), exponential with an intercept ($K_{leaf} = C + Ae^{-B\Psi_{leaf}}$), logistic ($K_{leaf} = \frac{A}{1+\left(\frac{\Psi_{leaf}}{x_0}\right)^B}$) and sigmoidal ($K_{leaf} = \frac{A}{1+e^{-\left(\frac{\Psi_{leaf}-x_0}{B}\right)}}$) (Sack & Scoffoni, 2012). The differences among AICc values for these functions were not large within species. We chose the exponential function without the intercept to fit observed data across species, and as a general function to scale-up to the community, because it had the lowest AICc for most species, captured the form of the observed data across all species well, and had the lowest number of parameters, that is, two.

Dataset S3 Wood specific gravity (WSG) and leaf mass area (LMA).

We used WSG, the wood specific gravity after drying at 100∘C, and LMA, the leaf mass per unit area measured for the leaf lamina excluding the petiole and for compound leaves the petiolules for leaves receiving direct sunlight as described in Wright *et al.* (2010).

Among the 21 species with observed data for (K_{leaf} vs. Ψ_{leaf}) used to build the community-level models of leaf vulnerability curves, two lacked data for WSG and five in LMA. We filled three gaps in LMA by substituting species-level means for $\mathrm{LMA}_{\mathrm{disc}}$, the latter defined as the mean leaf mass per unit area measured for a 1.483 cm^2 leaf disc taken to avoid veins (g m^{-2}) for leaves receiving direct sunlight, in a linear relationship between LMA and $\mathrm{LMA}_{\mathrm{disc}}$ ($\overline{\mathrm{LMA}} = -2.25 + 1.05\mathrm{LMA}_{disc}$; Adj. $R^2 = 0.87$; $p < 0.001$, $n = 202$). We filled the two remaining gaps in LMA by substituting LMA measured during the K_{leaf} campaign, $\mathrm{LMA}_{\mathrm{campaign}}$, in a linear relationship between LMA and ($\overline{\mathrm{LMA}} = 25.87 + 0.97\mathrm{LMA}_{campaign}$; Adj. $R^2 = 0.59$; $p < 0.001$, $n = 16$). We chose not to fill the two data gaps in WSG as available data were poor predictors of WSG.

Dataset S4 Leaf deciduousness categories.

At Barro Colorado Island SJW and Osvaldo Calderón have drawn on 62+ years of experience in Panama to score each species as one among four leaf habits (Meakem *et al.*, 2018): evergreen species (n = 17, for species with ERD) keep most but not all of their canopy in the dry season; obligate deciduous species (n = 25) shed all their leaves in the beginning of the dry season; facultative deciduous (n = 22) species drop leaves steadily as the dry season develops, and both facultative and obligate deciduous species leaf out in the beginning of the wet season; brevi-deciduous species (n = 23) shed all their leaves in the dry season, but leaf out in a few days to six weeks under ongoing dry season.

Dataset S5 Stream discharge from the Conrad catchment.

The Conrad weir is located on the gently sloping western side of Barro Colorado Island. The weir consists of a 90 degree 'V' notch with a rectangular upper section. The weir was constructed in several stages between 1993 and 1996. Stream stage is recorded at 5-minute intervals. The weir drains a catchment of approximately 40 ha of which at least 90% consists of the central plateau area of the island. Peak flow usually follows peak rain by approximately 2-3 hours. Large storms are sometimes preceded by small, initial peaks generated by the rain falling on the steeper slopes close to the weir. Streamflow data are analyzed by software custom-built by author SRP. The program is used to correct for blockages of the weir 'V' by fallen vegetation (a common occurrence), sensor drift, small gaps and other problems. Discharge is then calculated using an empirically derived rating curve calculated for each 1mm stream stage.

Dataset S6 Volumetric water content.

We collected volumetric water content (VWC) data (2012-2018) from three locations near the eddy flux tower with time domain reflectometry (TDR, CS616, Campbell Scientific) probes inserted vertically ranging over the first 15 cm of soil. The relationship between the output from the TDR probe and VWC was calibrated based on measurements of gravimetric water content (GWC) on several occasions from representative soils near the vertical probes (0-0.15 m) (Fig. S4). GWC was converted to VWC based on the observed mean bulk dry density of 0.8 g cm^{-3} near the TDR probe (n = 5, cylinder volume = 1250 cm^3). Beginning in 2016 we additionally installed three horizontal probes at depths of 0.15, 0.4 and 1 m and we applied the same calibration equation to these data (2016-2018) as that for the vertical probe. These data are archived at https://ngt-data.lbl.gov/ (DOI pending).

We also leveraged existing GWC measurements by Kupers *et al.* (2019) at the depths of 0.15, 0.4 and 1 m in summer 2015 (February, March and April) and 0.15, 0.4 in summer 2016 (March), with 1299 samples in total that covered all soil types and habitats in the 50ha plot. The sampling periods were six, five, ten and eight days long, respectively. The 2016 dry season was associated with the 2015–2016 El Niño (see Kupers *et al.* (2019) for further details). We converted these GWC measurements to VWC assuming a bulk density of 0.8 g cm^{-3}. $\theta_{0.15\ m}$, $\theta_{0.4\ m}$ and $\theta_{1\ m}$ refer to the plot-wide VWC averages by depth over each sampling period. Middle date of a sampling period is used for comparison with model simulations.

Dataset S7 Stem maximum hydraulic conductivity and vulnerability to cavitation.

Values of stem maximum hydraulic conductivity and vulnerability to cavitation were obtained from the dataset of Wolfe *et al.* (2021). The entire dataset includes measurements on 26 tree species collected at three sites in Panama. The raw data, *R* script for the analyses, and data summaries are archived in the NGEE-Tropics archive (Wolfe *et al.*, 2021). The data were previously reported by Dickman *et al.* (2019) and Wu *et al.* (2020). Here, the seven species that overlapped with our assessment of ERD were included in analyses (Table S3).

Methods for stem maximum hydraulic conductivity and vulnerability to cavitation closely followed Christman *et al.* (2012) and Wolfe *et al.* (2016). Briefly, canopy branches were collected from 4–10 trees of each species. Segments measured for hydraulic conductivity were removed from the branches and placed in an apparatus similar to that described by Sperry *et al.* (1988) except that flow rate was measured with graduated pipettes instead of a balance. Each segment was perfused with a 10 mM KCl solution filtered to 0.2 mM at three pressure heads: two that ranged 0.5–2 kPa and at zero pressure. Following Torres-Ruiz *et al.* (2012), hydraulic conductivity was calculated as the slope of the flow rate versus pressure gradient (i.e., pressure head divided by segment length) among the three pressure heads. Stem area specific hydraulic conductivity (K_{stem}) was calculated by dividing hydraulic conductivity by the cross-sectional area of the stem. For each K_{stem} segment, three measurements of stem water potential were made on stem sections that were located adjacent to the K_{stem} segment using psychrometers as described by Wolfe (2017).

To assess vulnerability to cavitation, the branches were allowed to dry in the lab for 0 – 180 hours before they were measured for stem water potential and K_{stem}. For each species, K_{stem} was plotted against stem water potential and a Weibull curve was fit through the 90th percentile of the points, following Wolfe *et al.* (2016). The intercept of the curve was interpreted as maximum K_{stem} and the water potential where the Weibull curve predicted 88% loss of maximum K_{stem} was interpreted as $\Psi_{88,stem}$.

Dataset S8 Leaf turgor loss point.

For each tree species measured for K_{stem} and $\Psi_{88,stem}$, two to six leaves were measured for Ψ_{tlp} with pressure-volume analysis following Koide *et al.* (1989). Leaf water potential was measured on leaf discs with psychrometers (J.R.D. Merrill Specialty Equipment, Logan, Utah, USA). This method has been shown to give results similar to those of the pressure chamber method (Nardini *et al.*, 2008). To construct the pressure-volume curves, leaf discs were repeatedly measured for water potential and mass. Between measurements the discs were bench dried for 5-30 minutes. Dry mass was determined after oven drying the discs at 70°C.

Dataset S9 Above-ground hydraulic safety margins.

Above-ground hydraulic safety margin was calculated as the difference between minimum leaf water potential Ψ_{min} and $\Psi_{88,stem}$. Values for Ψ_{min} were obtained from the dataset of Wolfe *et al.* (2019), which was collected as part of the 2016 ENSO campaign. Pre-dawn and diurnal leaf water potentials were measured on a monthly basis from Feb to May 2016 at San

Lorenzo and Parque Natural Metropolitano, Panama, for which leaves were sampled using a canopy crane. Measurements in BCI were available only for March 2016, for which leaves were sampled with a pole pruner, as there is no canopy crane on BCI. In total 25 species were sampled, with no species re-sampled on another site. The raw data and data summaries are archived in the NGEE-Tropics archive (Wolfe *et al.*, 2019). Here, the six species that overlapped with our assessment of ERD and $\Psi_{88,stem}$ were included in analyses. We defined species Ψ_{min} as the minimum of all the diurnal measurements for a species after taking an average value of measurements on two leaf samples for a given diurnal measurement (Table S3).

Notes S1 ERD model structure selection.

The best ERD model structure explained a large fraction of the variance in δ^2H_{xylem}. This model included an effect of VPD and not LAI (Eq. (S3)). Two of the alternative models (Eqs. (S4) & (S6)) with LAI had a strong relationship with δ^2H_{xylem} as well, but one that was based on a smaller set of species (n = 4 or 5), because fewer species passed the criteria of sufficient species-level goodness-of-fit for these models (<17 species vs. 29 species for the best model, Fig. S11). For the rest of the analyses, we therefore used ERD estimates from the less parsimonious model (Eq. (S3)).

Notes S2 Additional results for exposure to water-stress.

Overall, species with ERD deeper than 2.9 m did not cross species Ψ_{crit} for the entire 25-yr period for which $\Psi_{soil,z}$ was modeled (1990-2015). Soil depths deeper than 2.9 m remained above -0.06 MPa (-0.02, -0.14; median, 95% CI) through all the dry seasons and drought periods, which is above Ψ_{crit} of the most sensitive tree species (-0.17 MPa).

References

Ali AA, Xu C, Rogers A, McDowell NG, Medlyn BE, Fisher RA, Wullschleger SD, Reich PB, Vrugt JA, Bauerle WL *et al.* 2015. Global-scale environmental control of plant photosynthetic capacity. *Ecological Applications* **25**: 2349–2365.

Broedel E, Tomasella J, Cândido LA, Randow C von. **2017**. Deep soil water dynamics in an undisturbed primary forest in central Amazonia: Differences between normal years and the 2005 drought. *Hydrological Processes* **31**: 1749–1759.

Campbell GS. **1974**. A simple method for determining unsaturated conductivity from moisture retention data. *Soil Science* **117**: 311–314.

Canadell J, Jackson RB, Ehleringer JB, Mooney HA, Sala OE, Schulze ED. **1996**. Maximum rooting depth of vegetation types at the global scale. *Oecologia* **108**: 583–595.

Christman MA, Sperry JS, Smith DD. **2012**. Rare pits, large vessels and extreme vulnerability to cavitation in a ring-porous tree species. *New Phytologist* **193**: 713–720.

Condit R. **2017**. Demographic trends and climate over 35 years in the Barro Colorado 50 ha plot. *Forest Ecosystems* **4**: 1–13.

Condit R, Pérez R, Aguilar S, Lao S, Foster R, Hubbell S. **2019**. Complete data from the Barro Colorado 50-ha plot: 423617 trees, 35 years, v3, DataONE, Dataset, https://doi.org/10.15146/5xcp-0d46.

Condit R, Watts K, Bohlman SA, Pérez R, Foster RB, Hubbell SP. **2000**. Quantifying the deciduousness of tropical forest canopies under varying climates. *Journal of Vegetation Science* **11**: 649–658.

Davidson E, Lefebvre PA, Brando PM, Ray DM, Trumbore SE, Solorzano LA, Ferreira JN, Bustamante MM da C, Nepstad DC. **2011**. Carbon Inputs and Water Uptake in Deep Soils of an Eastern Amazon Forest. *Forest Science* **57**: 51–58.

Detto M, Baldocchi D, Katul GG. **2010**. Scaling Properties of Biologically Active Scalar Concentration Fluctuations in the Atmospheric Surface Layer over a Managed Peatland. *Boundary-Layer Meteorology* **136**: 407–430.

Dickman LT, McDowell NG, Grossiord C, Collins AD, Wolfe BT, Detto M, Wright SJ, Medina-Vega JA, Goodsman D, Rogers A *et al.* 2019. Homoeostatic maintenance of nonstructural carbohydrates during the 2015–2016 El Niño drought across a tropical forest precipitation gradient. *Plant, Cell & Environment* **42**: 1705–1714.

Godsey S, Elsenbeer H, Stallard R. **2004**. Overland flow generation in two lithologically distinct rainforest catchments. *Journal of hydrology* **295**: 276–290.

Hagan MT, Menhaj MB. **1994**. Training feedforward networks with the Marquardt algorithm. *IEEE Transactions on Neural Networks* **5**: 989–993.

Kinner DA, Stallard RF. **2004**. Identifying storm flow pathways in a rainforest catchment using hydrological and geochemical modelling. *Hydrological Processes* **18**: 2851–2875.

Koide RT, Robichaux RH, Morse SR, Smith CM. **1989**. Plant water status, hydraulic resistance and capacitance. In: Pearcy R, Ehleringer J, Mooney H, Rundel P, eds. Plant physiological ecology: Field methods and instrumentation. London: Chapman; Hall, 161–183.

Kupers SJ, Wirth C, Engelbrecht BMJ, Rüger N. **2019**. Dry season soil water potential maps of a 50 hectare tropical forest plot on Barro Colorado Island, Panama. *Scientific data* **6**: 63.

Lasslop G, Reichstein M, Papale D, Richardson A, Arneth A, Barr A, Stoy P, Wohlfahrt G. **2010**. Separation of net ecosystem exchange into assimilation and respiration using a light response curve approach: critical issues and global evaluation. *Global Change Biology* **16**: 187–208.

Meakem V, Tepley AJ, Gonzalez-Akre EB, Herrmann V, Muller-Landau HC, Wright SJ, Hubbell SP, Condit R, Anderson-Teixeira KJ. **2018**. Role of tree size in moist tropical forest carbon cycling and water deficit responses. *New Phytologist* **219**: 947–958.

Medlyn BE, Duursma RA, Eamus D, Ellsworth DS, Prentice IC, Barton CVM, Crous KY, Angelis P de, Freeman M, Wingate L. **2012**. Reconciling the optimal and empirical approaches to modelling stomatal conductance. *Global Change Biology* **18**: 3476–3476.

Meinzer FC, Andrade JL, Goldstein G, Holbrook NM, Cavelier J, Wright SJ. **1999**. Partitioning of soil water among canopy trees in a seasonally dry tropical forest. *Oecologia* **121**: 293–301.

Muller-Landau HC, Condit RS, Chave J, Thomas SC, Bohlman SA, Bunyavejchewin S, Davies S, Foster R, Gunatilleke S, Gunatilleke N *et al.* 2006. Testing metabolic

ecology theory for allometric scaling of tree size, growth and mortality in tropical forests. *Ecology Letters* **9**: 575–588.

Nardini A, Gortan E, Ramani M, Salleo S. **2008**. Heterogeneity of gas exchange rates over the leaf surface in tobacco: an effect of hydraulic architecture? *Plant, Cell & Environment* **31**: 804–812.

Nepstad DC, Carvalho CR de, Davidson EA, Jipp PH, Lefebvre PA, Negreiros GH, Silva ED da, Stone TA, Trumbore SE, Vieira S. **1994**. The role of deep roots in the hydrological and carbon cycles of Amazonian forests and pastures. *Nature* **372**: 666–669.

Oleson K, Bonan GB, Drewniak B, Huang M, Koven CD, Levis S, Lawrence DM, Li F, Riley WJ, Subin ZM *et al.* 2013. Technical description of version 4.5 of the Community Land Model (CLM) (No. NCAR/TN-503+STR). doi:10.5065/D6RR1W7M.

Osnas JLD, Katabuchi M, Kitajima K, Wright SJ, Reich PB, Bael SAV, Kraft NJB, Samaniego MJ, Pacala SW, Lichstein JW. **2018**. Divergent drivers of leaf trait variation within species, among species, and among functional groups. *Proceedings of the National Academy of Sciences* **115**: 201803989.

Papale D, Valentini R. **2003**. A new assessment of European forests carbon exchanges by eddy fluxes and artificial neural network spatialization. *Global Change Biology* **9**: 525–535.

Paton S. **2019**. Barro Colorado Island, Conrad Catchment_Runoff. Dataset. The Smithsonian Institution. Accessed at https://doi.org/10.25573/data.10042478.v2.

Pau S, Detto M, Kim Y, Still CJ. **2018**. Tropical forest temperature thresholds for gross primary productivity. *Ecosphere* **9**: e02311.

Sack L, Bartlett M, Creese C, Guyot G, Scoffoni C. **2011**. Constructing and operating a hydraulics flow meter. *PrometheusWiki*.

Sack L, Scoffoni C. **2012**. Measurement of Leaf Hydraulic Conductance and Stomatal Conductance and Their Responses to Irradiance and Dehydration Using the Evaporative Flux Method (EFM). doi: 10.3791/4179. *Journal of Visualized Experiments : JoVE*: 4179.

Sperry JS, Donnelly JR, Tyree MT. **1988**. A method for measuring hydraulic conductivity and embolism in xylem. *Plant, Cell & Environment* **11**: 35–40.

Tomasella J, Hodnett MG. **1998**. Estimating soil water retention characteristics from limited data in Brazilian Amazonia. *Soil Science* **163**: 190–202.

Torres-Ruiz JM, Sperry JS, Fernández JE. **2012**. Improving xylem hydraulic conductivity measurements by correcting the error caused by passive water uptake. *Physiologia Plantarum* **146**: 129–135.

Wolfe BT. **2017**. Retention of stored water enables tropical tree saplings to survive extreme drought conditions. *Tree Physiology* **37**: 469–480.

Wolfe BT, Cozzarelli Y, Jaén N. **2021**. Stem hydraulic conductivity and vulnerability to cavitation for 26 tree species in Panama. 1.0. NGEE Tropics Data Collection. http://dx.doi.org/10.15486/ngt/1770723.

Wolfe BT, Sperry JS, Kursar TA. **2016**. Does leaf shedding protect stems from cavitation during seasonal droughts? A test of the hydraulic fuse hypothesis. *New Phytologist* **212**: 1007–1018.

Wolfe BT, Wu J, Ely K, Serbin S, Rogers A, Dickman T, Collins A, Detto M, Grossiord C, McDowell N *et al.* 2019. Leaf water potential, Feb2016-May2016, PA-SLZ, PA-PNM,

PA-BCI: Panama. 1.0. NGEE Tropics Data Collection. Accessed at http://dx.doi.org/10.15486/ngt/1507766.

Wright SJ, Cornejo FH. **1990**. Seasonal Drought and Leaf Fall in a Tropical Forest. *Ecology* **71**: 1165–1175.

Wright SJ, Kitajima K, Kraft NJB, Reich PB, Wright IJ, Bunker DE, Condit R, Dalling JW, Davies SJ, Díaz S *et al.* 2010. Functional traits and the growth–mortality trade-off in tropical trees. *Ecology* **91**: 3664–3674.

Wu J, Serbin SP, Ely KS, Wolfe BT, Dickman LT, Grossiord C, Michaletz ST, Collins AD, Detto M, McDowell NG *et al.* 2020. The response of stomatal conductance to seasonal drought in tropical forests. *Global Change Biology* **26**: 823–839.

Zeng X. **2001**. Global Vegetation Root Distribution for Land Modeling. *Journal of Hydrometeorology* **2**: 525–530.

Zimmermann B, Zimmermann A, Lark RM, Elsenbeer H. **2010**. Sampling procedures for throughfall monitoring: A simulation study. *Water Resources Research* **46**: W01503.
